# BRD4 inhibition exerts anti-viral activity through DNA damage-dependent innate immune responses

**DOI:** 10.1371/journal.ppat.1008429

**Published:** 2020-03-24

**Authors:** Jiang Wang, Guo-Li Li, Sheng-Li Ming, Chun-Feng Wang, Li-Juan Shi, Bing-Qian Su, Hong-Tao Wu, Lei Zeng, Ying-Qian Han, Zhong-Hu Liu, Da-Wei Jiang, Yong-Kun Du, Xiang-Dong Li, Gai-Ping Zhang, Guo-Yu Yang, Bei-Bei Chu

**Affiliations:** 1 College of Animal Sciences and Veterinary Medicine, Henan Agricultural University, Zhengzhou, Henan Province, P.R. China; 2 Jiangsu Co-innovation Center for Prevention and Control of Important Animal Infectious Diseases and Zoonoses, College of Veterinary Medicine, Yangzhou University, Jiangsu Province, P.R. China; University of Chicago, UNITED STATES

## Abstract

Chromatin dynamics regulated by epigenetic modification is crucial in genome stability and gene expression. Various epigenetic mechanisms have been identified in the pathogenesis of human diseases. Here, we examined the effects of ten epigenetic agents on pseudorabies virus (PRV) infection by using GFP-reporter assays. Inhibitors of bromodomain protein 4 (BRD4), which receives much more attention in cancer than viral infection, was found to exhibit substantial anti-viral activity against PRV as well as a range of DNA and RNA viruses. We further demonstrated that BRD4 inhibition boosted a robust innate immune response. BRD4 inhibition also de-compacted chromatin structure and induced the DNA damage response, thereby triggering the activation of cGAS-mediated innate immunity and increasing host resistance to viral infection both *in vitro* and *in vivo*. Mechanistically, the inhibitory effect of BRD4 inhibition on viral infection was mainly attributed to the attenuation of viral attachment. Our findings reveal a unique mechanism through which BRD4 inhibition restrains viral infection and points to its potent therapeutic value for viral infectious diseases.

## Author summary

BRD4 has been well investigated in tumorigenesis for its contribution to chromatin remodeling and gene transcription. BRD4 inhibitors are used as promising chemotherapeutic drugs for cancer therapy. Here, we show a unique mechanism through which BRD4 inhibition broadly inhibits attachment of DNA and RNA viruses through DNA damage-dependent antiviral innate immune activation *via* the cGAS-STING pathway, in both cell culture and an animal model. STING-associated innate immune signaling has been considered to be a new possibility for cancer therapy, and STING agonists have been tested in early clinical trials. Our data identify BRD4 inhibitors as a potent therapy not only for viral infection but also for cancer immunotherapy.

## Introduction

Epigenetic modulation of the structure of chromatin, including DNA modifications and post-translational modifications of histones, is critical for the regulation of gene expression [1, 2]. Many enzymes involved in epigenetic modulation of chromatin have been identified. These include DNA methyltransferases and DNA demethylases; histone acetyltransferases and histone deacetylases; and lysine methyltransferases and lysine demethylases. DNA methylation regulates gene expression by recruiting proteins involved in gene repression or by inhibiting the binding of transcription factors [3]. Histone acetylation influences histone/DNA interactions in the nucleosome and perturbs histone/histone interactions [4]. Acetyl groups can also serve as a platform for recruitment of histone acetylation readers to participate in gene transcription, DNA replication, DNA repair or chromatin condensation [5]. Histone lysine methylation on histones H3 and H4 has been implicated in heterochromatin formation and the regulation of promoter activity [6, 7]. Dysregulation of epigenetic modifications is associated with various human diseases, such as cancer and neurodevelopmental disorders [8, 9].

Bromodomain protein 4 (BRD4) is a reader and writer of histone acetylation that plays important roles in replication, transcription and DNA repair [10, 11]. The post-translational modification of histone acetylation is a key mechanism that regulates chromatin organization, and several studies have focused on the important function of BRD4 in regulating chromatin structure [12–15]. The histone acetyltransferase activity of BRD4 is responsible for maintaining normal chromatin structure [16]. BRD4 is critical in the maintenance of higher-order chromatin structure, and inhibition of BRD4 leads to chromatin decondensation and fragmentation [17]. Another study has demonstrated that a short isoform of BRD4 lacking the histone acetyltransferase domain can recruit the condensing II remodeling complex, thus forming a closed chromatin structure [18]. Otherwise, BRD4 can de-compact chromatin and facilitate transcriptional re-activation [19]. BRD4 acetylates histone H3 at the K122 residue, thereby perturbing a salt bridge and leading to nucleosome instability [16]. Thus, the mechanism by which BRD4 contributes to chromatin structure is likely to be complex and context-specific.

Detection of double-stranded DNA (dsDNA) in the cytosol by germline-encoded DNA sensors is a central mechanism of innate immune defense against infection in most organisms [20]. Cyclic GMP-AMP synthase (cGAS) is a predominant and general sensor of cytosolic DNA [21]. Upon binding of cGAS to dsDNA in the cytosol, cGAS enzymatic activity triggers the generation of 2′,3′-cyclic GMP-AMP (2′3′-cGAMP) from GTP and ATP [21, 22]. Stimulator of interferon genes (STING) binds 2′3′-cGAMP and undergoes a large conformational change [23, 24], thus enabling the recruitment of TANK binding kinase (TBK1) to STING; TBK1 further phosphorylates interferon (IFN)-regulated factor 3 (IRF3) and nuclear factor-κB, thus resulting in the expression of type I IFNs and proinflammatory cytokines [25]. Damage-associated cytosolic dsDNA released from the mitochondria or nucleus also activates innate immunity through the cGAS/STING/TBK1/IRF3 signaling pathway [26–29]. At present, no reports have suggested that BRD4 is involved in antiviral innate immunity.

Here, we describe a mechanism through which BRD4 inhibition stimulates antiviral innate immunity. We demonstrate that BRD4 inhibition exhibits broad-spectrum antiviral activity. BRD4 inhibition induces the DNA damage response (DDR), which in turn activates the cGAS/STING/TBK1/IRF3 innate immune pathway and inhibits viral attachment.

## Results

### Examination of the antiviral activities of epigenetic agents *in vitro*

**Fig 1:**
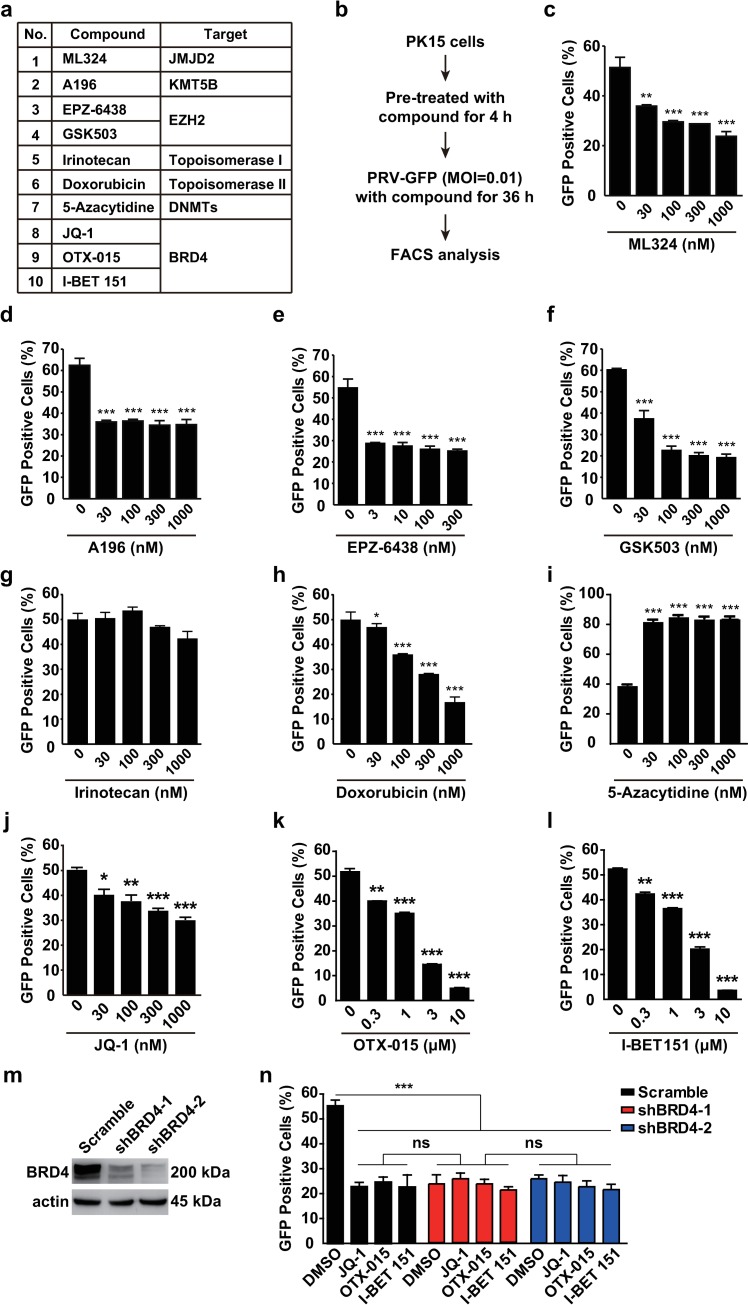
Examination of potent antivirals with GFP-reporter assays. (a) A list of ten epigenetic agents. (b) Schematic for GFP-reporter assays. PK15 cells were seeded in 24-well plates. After the cells were cultured to approximately 60%–70% confluence, the compounds were added to cells for 4 h at 37°C. Then cells were incubated with PRV-GFP (MOI = 0.01) and different concentrations of compounds for another 36 h at 37°C. Flow cytometry analysis was carried out to examine the GFP fluorescence representing viral replication. (c-l) Results of flow cytometry assays with small-molecule inhibitors listed in a. Data are shown as mean ± SD based on three independent experiments. * P < 0.05, ** P < 0.01, *** P < 0.001 determined by two-tailed Student’s *t-*test. (m) BRD4 was assessed with immunoblotting in Scramble, shBRD4-1 and shBRD4-2 PK15 cells. Actin served as a loading control. (n) PRV-GFP proliferation was assessed by flow cytometry in Scramble, shBRD4-1 and shBRD4-2 PK15 cells infected with PRV-GFP (MOI = 0.01) and treated with DMSO, JQ-1 (1 μM), OTX-015 (10 μM) and I-BET 151 (10 μM) for 36 h. Data are shown as mean ± SD based on three independent experiments. * P < 0.05, ** P < 0.01, *** P < 0.001 determined by one-way ANOVA. ns, no significance.

We attempted to examine the roles of epigenetic agents targeting histone modifiers and chromatin remodelers, including Jumonji domain 2 containing histone demethylase (JMJD2), lysine methyltransferase 5B, enhancer of zeste 2 polycomb repressive complex 2 subunit, DNA topoisomerase, DNA methyltransferases and BRD4 in viral infection (Fig 1A). We used a recombinant pseudorabies virus expressing a GFP reporter (PRV-GFP) to assess viral replication under treatment with these inhibitors *in vitro* (Fig 1B). The inhibitor of JMJD2, ML324, which has been reported to have potent anti-viral activity against both herpes simplex virus (HSV) and human cytomegaloviral infection [30], also showed a significant inhibitory effect toward PRV-GFP infection (Fig 1C). Inhibition of lysine methyltransferase by A196, EPZ-6438 and GSK503 decreased the rate of PRV-GFP-positive cells, thus suggesting that histone methylation is essential for PRV infection (Fig 1D–1F). In addition, PRV-GFP replication declined in cells treated with the DNA topoisomerase II inhibitor doxorubicin but not the DNA topoisomerase I inhibitor irinotecan (Fig 1G and 1H). The inhibitor of DNA methyltransferases 5-Azacytidine inhibits human immunodeficiency virus type 1 replication *in vitro* [31], whereas we found that treatment with 5-Azacytidine markedly promoted PRV-GFP replication (Fig 1I).

Three inhibitors against BRD4, JQ-1, OTX-015 and I-BET 151, exhibited viral inhibition upon PRV-GFP infection (Fig 1J–1L). Because acetylation is critical in histone modification, we next sought to identify the mechanisms through which BRD4 inhibition suppresses viral infection. To validate the specificity of BRD4 inhibitors, we depleted BRD4 with a short hairpin RNA (shRNA; shBRD4) in PK15 cells (Fig 1M). Knockdown of BRD4 also decreased the rate of PRV-GFP-positive cells (Fig 1N). Treatment of BRD4 depleted cells with JQ-1, OTX-015 and I-BET 151 showed no cumulative effect on PRV-GFP infection, thus suggesting that BRD4 inhibitors specifically acted on BRD4 to reduce PRV infection (Fig 1N).

### Effects of BRD4 inhibition on cell viability, cell-cycle arrest and apoptosis

**Fig 2:**
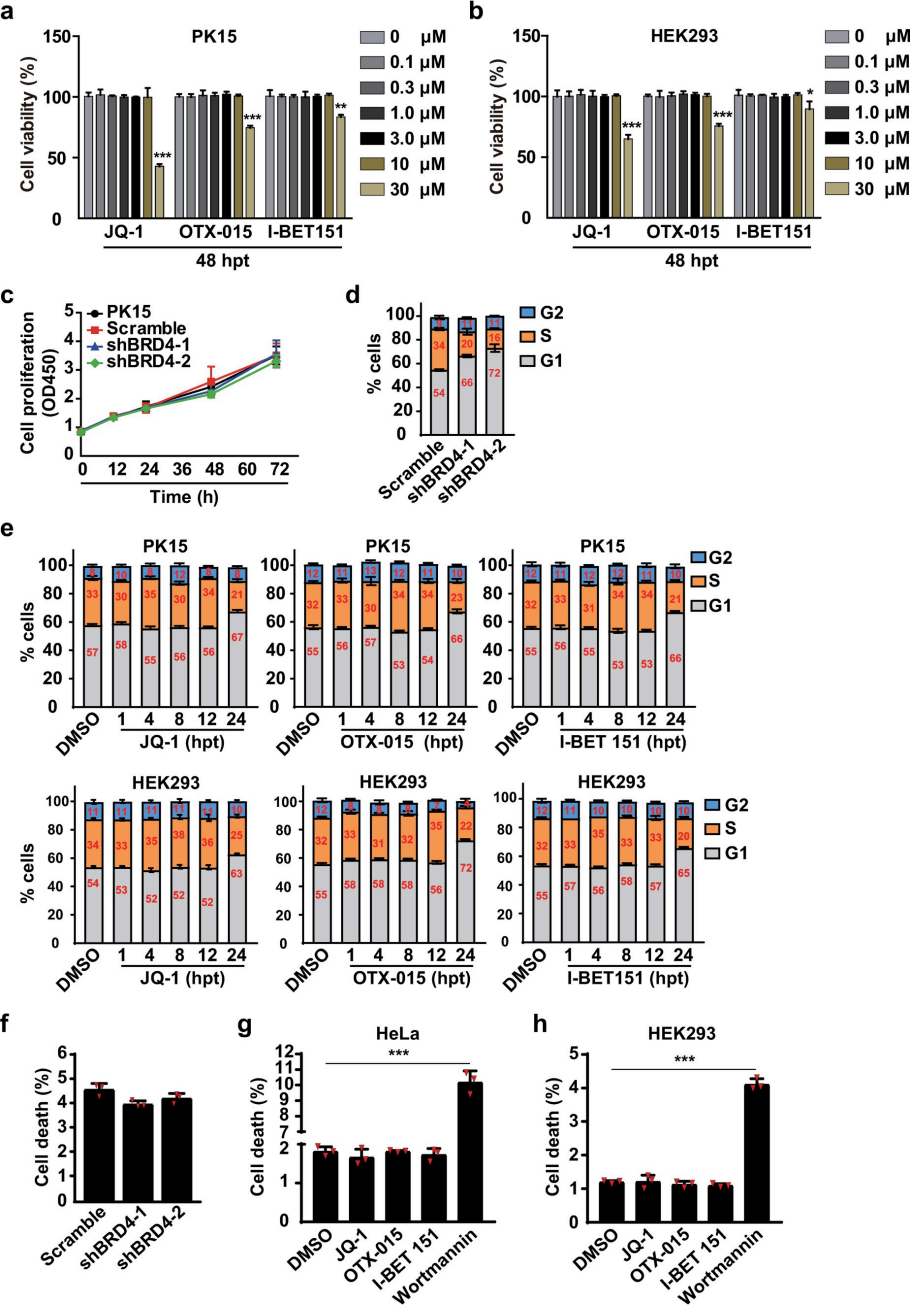
BRD4 inhibition induces cell cycle arrest but not apoptosis. (a and b) Cell viability was assessed with CCK-8 cell counting assays in PK15 and HEK293 cells treated with 0–30 μM of JQ-1, OTX-015 and I-BET 151 at 48 hpt. Data are shown as mean ± SD based on three independent experiments. * P < 0.05, ** P < 0.01, *** P < 0.001 determined by two-tailed Student’s *t*-test. (c) Cell proliferation was assessed with CCK-8 cell counting assays in Scramble, shBRD4-1 and shBRD4-2 PK15 cells cultured for 0, 12, 24, 36, 48, 60 and 72 h. (d) The cell cycle was assessed with flow cytometry in Scramble, shBRD4-1 and shBRD4-2 PK15 cells cultured for 24 h. (e) The cell cycle was assessed with flow cytometry in PK15 and HEK293 cells treated with DMSO, JQ-1 (1 μM), OTX-015 (10 μM) and I-BET 151 (10 μM) at 1, 4, 8, 12 and 24 hpt. (f) Apoptosis was assessed with flow cytometry in Scramble, shBRD4-1 and shBRD4-2 PK15 cells cultured for 24 h. (g and h) Apoptosis was assessed with flow cytometry in HeLa (g) and HEK293 (h) cells treated with DMSO, JQ-1 (1 μM), OTX-015 (10 μM), I-BET 151 (10 μM) and wortmannin (2.5 μM) for 24 h. Data are shown as mean ± SD based on three independent experiments. *** P < 0.001 determined by two-tailed Student’s *t*-test.

We then performed cell viability assays to examine the cytotoxicity of BRD4 inhibitors. Treatment with 0–10 μM of JQ-1, OTX-015 and I-BET 151 at 48 h post-treatment (hpt) was harmless both to PK15 and HEK293 cells (Fig 2A and 2B). Cytotoxicity was observed when the cells were treated with 30 μM of BRD4 inhibitors at 48 hpt (Fig 2A and 2B). The proliferation rate of PK15, scrambled control shRNA (Scramble), shBRD4-1 and shBRD4-2 cells was nearly the same, thus suggesting that down-regulation of BRD4 did not influence cell growth (Fig 2C). Because BRD4 inhibitors induce apoptosis of some cancer cells [32–34], we then assessed cell cycle progression and apoptosis when BRD4 was inhibited. We found that cells expressing low levels of BRD4 accumulated in G1 phase (Fig 2D). Similar patterns of cell-cycle arrest in G1 phase were also observed when PK15 and HEK293 cells were incubated with BRD4 inhibitors up to 24 hpt (Fig 2E).

We next examined apoptosis in cells in response to BRD4 inhibition. Knockdown of BRD4 did not promote apoptosis in PK15 cells, as assessed with flow cytometry assays of Annexin V/PI (Fig 2F and S1A Fig). At 24 hpt, the pro-apoptosis compound wortmannin [35], but not BRD4 inhibitors, induced significant apoptosis in HeLa and HEK293 cells (Fig 2G and 2H, S1B Fig). Furthermore, BRD4 inhibitors induced no activation of P53, a master regulator of apoptosis [36] in HEK293 cells, as assessed with immunoblotting analysis of phosphorylation of P53 and its downstream effectors MDM2 and P21 (S1C Fig). These results indicate that although BRD4 inhibition induces cell-cycle arrest, the cells do not undergo apoptosis.

### BRD4 inhibition exhibits broad antiviral activity *in vitro*

**Fig 3:**
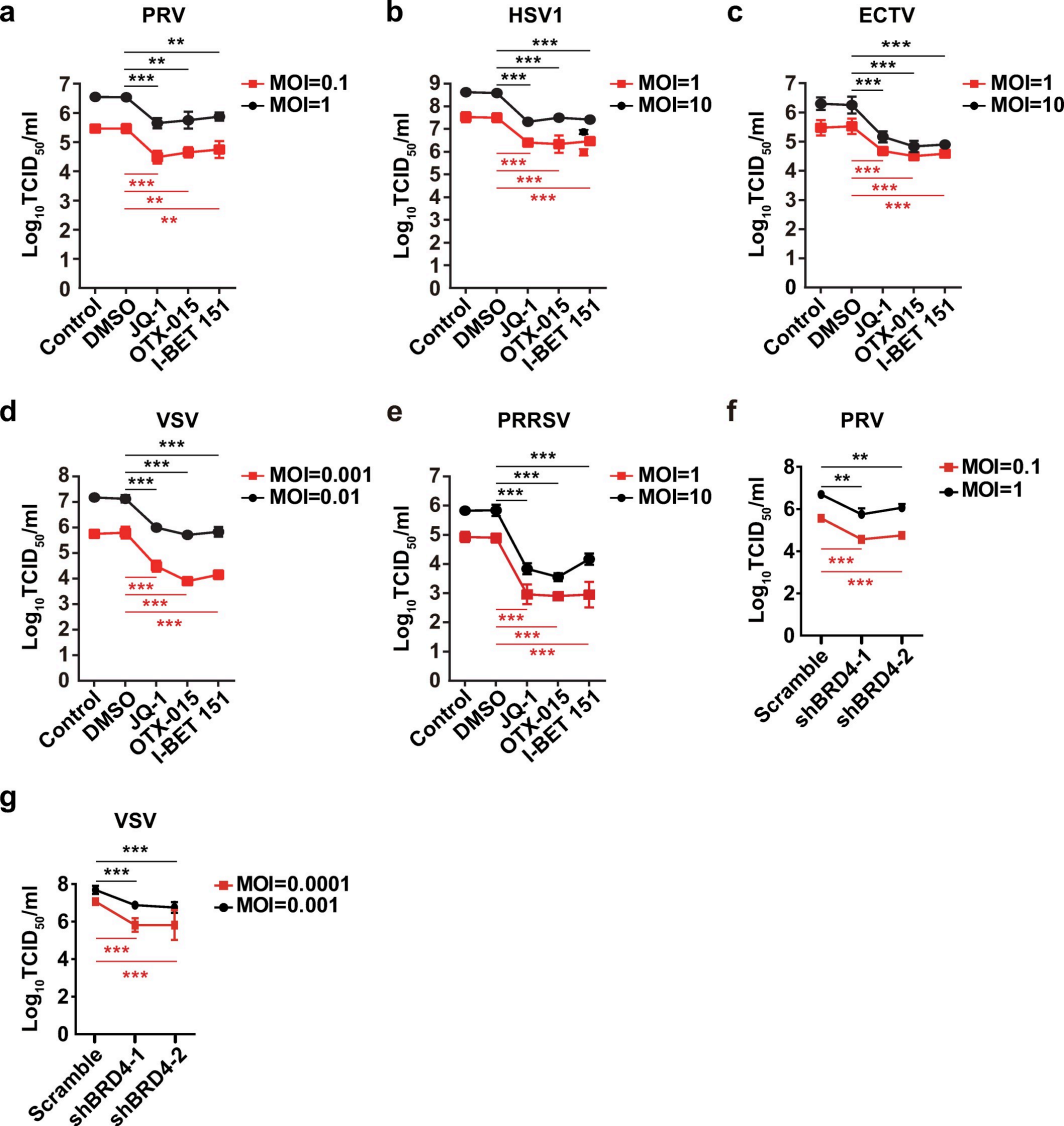
BRD4 inhibition exerts broad-spectrum antiviral activity. (a) Viral titer was assessed with TCID_50_ assays in PK15 cells (Control) infected with PRV-QXX (MOI = 0.1 and 1) and treated with DMSO, JQ-1 (1 μM), OTX-015 (10 μM) and I-BET 151 (10 μM) for 24 h. (b) Viral titer was assessed with TCID_50_ assays in A549 cells (Control) infected with HSV1-F (MOI = 1 and 10) and treated as in a. (c) Viral titer was assessed with TCID_50_ assays in Vero cells (Control) infected with ECTV (MOI = 1 and 10) and treated as in a. (d) Viral titer was assessed with TCID_50_ assays in PK15 cells (Control) infected with VSV-GFP (MOI = 0.001 and 0.01) and treated as in a. (e) Viral titer was assessed with TCID_50_ assays in MARC-145 cells (Control) infected with PRRSV-BJ4 (MOI = 1 and 10) and treated as in a. (f and g) Viral titer was assessed with TCID_50_ assays in Scramble, shBRD4-1 and shBRD4-2 PK15 cells infected with PRV-QXX (f, MOI = 0.1 and 1) and VSV-GFP (g, MOI = 0.001 and 0.01) for 24 h. All data are shown as mean ± SD based on three independent experiments. ** P < 0.01, *** P < 0.001 determined by two-tailed Student’s *t*-test.

We attempted to determine whether BRD4 inhibition might exhibit broad antiviral activity beyond that against PRV infection. We infected cells with PRV, HSV1, ectromelia virus (ECTV), vesicular stomatitis virus (VSV), porcine reproductive and respiratory syndrome virus (PRRSV), Newcastle disease virus (NDV) and influenza virus (H1N1) to examine the effects of BRD4 inhibition on virus replication by using viral titer assays. As expected, pharmacological inhibition of BRD4 by JQ-1, OTX-015 and I-BET 151 exerted anti-viral activities against DNA viruses, such as PRV, HSV1 and ECTV, in a dose-dependent manner (Fig 3A–3C and S2A–S2C Fig). Moreover, treatment of cells with BRD4 inhibitors decreased the replication of RNA viruses including VSV, PRRSV, NDV and H1N1 (Fig 3D and 3E, S2D–S2G Fig). We further determined the replication of PRV and VSV in Scramble, shBRD4-1 and shBRD4-2 PK15 cells. Knockdown of BRD4 caused significant attenuation of both PRV and VSV infection (Fig 3F and 3G). These data suggest that BRD4 inhibition promotes broad-spectrum antiviral activity.

### BRD4 inhibition does not impair the transcription of viral genes

**Fig 4:**
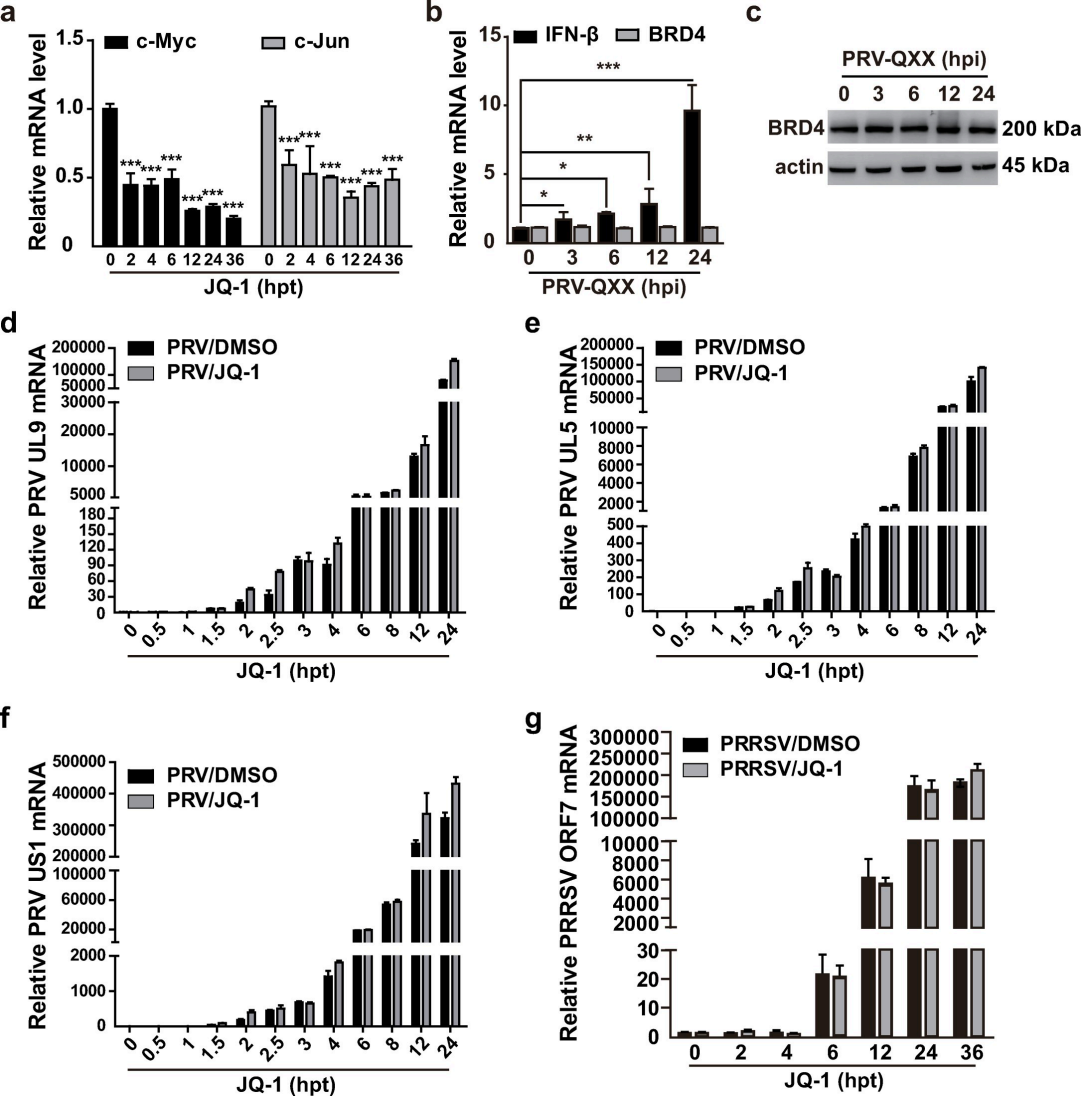
BRD4 inhibition does not impair the transcription of viral genes. (a) c-Myc and c-Jun mRNA were assessed with RT-qPCR analysis in PK15 cells treated with JQ-1 (1 μM) at 0–36 hpt. Data are shown as mean ± SD based on three independent experiments. *** P < 0.001 determined by two-tailed Student’s *t*-test. (b) IFN-β and BRD4 mRNA were assessed with RT-qPCR analysis in PK15 cells infected with PRV-QXX (MOI = 0.1) at 0–24 hpi. Data are shown as mean ± SD based on three independent experiments. * P < 0.05, ** P < 0.01, *** P < 0.001 determined by two-tailed Student’s *t*-test. (c) BRD4 expression was assessed with immunoblotting in PK15 cells infected with PRV-QXX (MOI = 0.1) at 0–24 hpi. Actin served as a loading control. (d-f) PRV UL9 (d), UL5 (e) and US1 (f) mRNA were assessed with RT-qPCR analysis in PK15 cells infected with PRV-QXX (MOI = 0.1) and treated with DMSO or JQ-1 (1 μM) at 0–24 hpt. (g) PRRSV ORF7 mRNA were assessed with RT-qPCR analysis in MARC-145 cells infected with PRRSV-BJ4 (MOI = 1) and treated with DMSO or JQ-1 (1 μM) at 0–36 hpt.

Given that BRD4 participates in gene transcription [37], we assessed whether BRD4 inhibition might perturb viral gene transcription and impair viral infection. In agreement with previous results, JQ-1 treatment dramatically suppressed the transcription of c-Myc and c-Jun in PK15 cells, both of which were regulated by BRD4 [38] (Fig 4A). We next sought to determine whether PRV infection might influence BRD4 expression at both the mRNA and protein levels. Although PRV infection elevated the expression of IFN-β mRNA [39], the mRNA and protein expression of BRD4 were normal at 0, 3, 6, 12 and 24 h post-infection (hpi, Fig 4B and 4C).

We then infected PK15 cells with PRV-QXX and simultaneously treated with JQ-1 for different lengths of time. RT-qPCR analysis indicated that JQ-1 treatment did not suppress the transcription of PRV genes, such as UL9, UL5 and US1 (Fig 4D–4F). We also detected PRRSV gene transcription after JQ-1 treatment. As with the results for PRV gene transcription, the mRNA of PRRSV ORF7 was not suppressed in MARC-145 cells treated with JQ-1 for 0–36 h (Fig 4G). These results demonstrate that BRD4 inhibition does not influence viral gene transcription.

### BRD4 inhibition attenuates viral attachment

**Fig 5:**
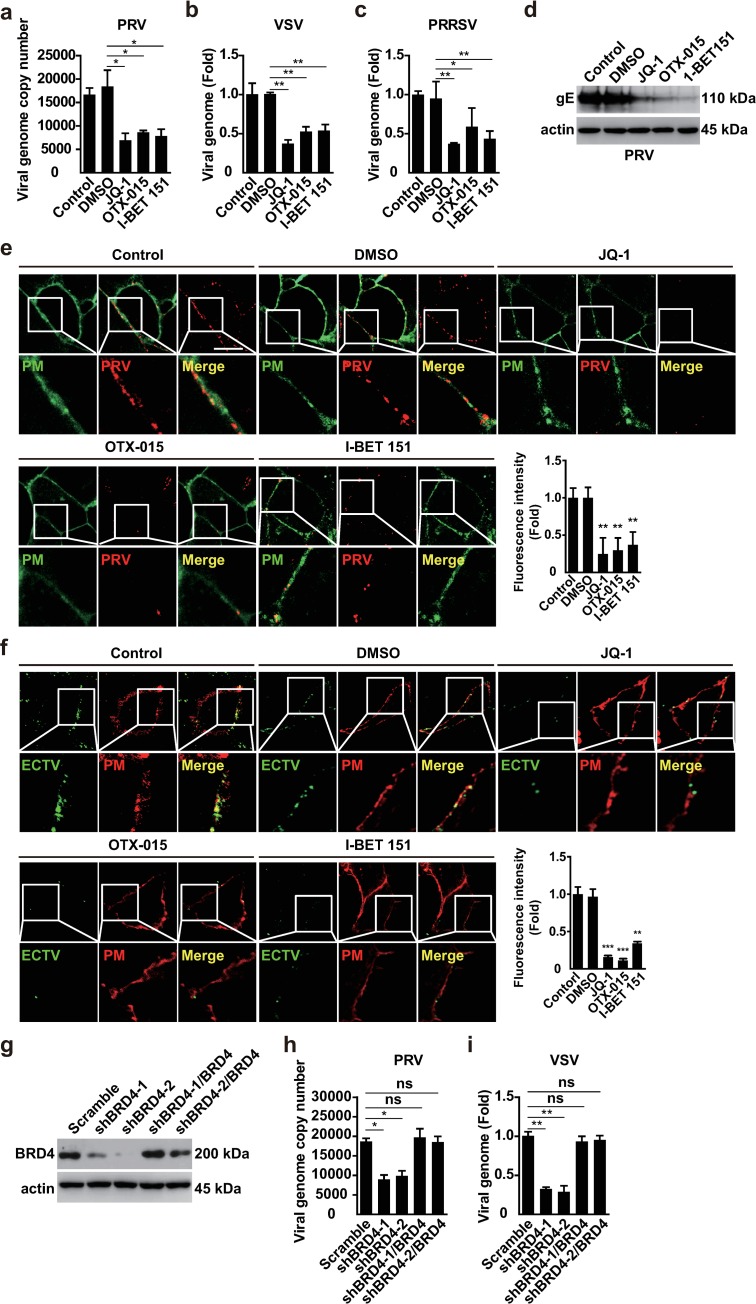
BRD4 inhibitors attenuate viral attachment. (a and b) Viral attachment was assessed with RT-qPCR analysis in PK15 cells incubated with PRV-QXX (a, MOI = 1) or VSV-GFP (b, MOI = 0.01). (c) Viral attachment was assessed with RT-qPCR analysis in MARC-145 cells incubated with PRRSV-BJ4 (MOI = 10). (d) Viral attachment was assessed with immunoblotting analysis against PRV gE in PK15 cells incubated with PRV-QXX (MOI = 1). Actin served as a loading control. (e) Viral attachment was assessed with fluorescence analysis in PK15 cells incubated with EdU-labeled PRV-QXX (MOI = 1) which were detected by Apollo staining (red). CellMask Green staining (green) indicated the plasma membrane. Scale bar, 10 μM. (f) Viral attachment was assessed with fluorescence analysis in Vero cells incubated with EdU-labeled ECTV (MOI = 10) which were detected by Apollo staining (green). CellMask Deep red staining (red) indicated the plasma membrane. Scale bar, 10 μM. (g) BRD4 expression was assessed with immunoblotting in Scramble, shBRD4-1, shBRD4-2, shBRD4-1/BRD4 and shBRD4-2/BRD4 PK15 cells. Actin served as a loading control. (h and i) Viral attachment was assessed with RT-qPCR analysis in Scramble, shBRD4-1, shBRD4-2, shBRD4-1/BRD4 and shBRD4-2/BRD4 PK15 cells incubated with PRV-QXX (h, MOI = 1) or VSV-GFP (i, MOI = 0.01). All data are shown as mean ± SD based on three independent experiments. * P < 0.05, ** P < 0.01, *** P < 0.001 determined by two-tailed Student’s *t*-test.

To determine how BRD4 inhibition influenced viral infection, we pre-treated PK15 cells with DMSO, JQ-1, OTX-015 and I-BET 151 for 24 h at 37°C. Then, the cells were incubated with PRV and BRD4 inhibitors for 1 h at 4°C. RT-qPCR analysis indicated that the viral genome copy number in control cells and cells treated with DMSO was greater than that in cells treated with JQ-1, OTX-015 and I-BET 151, thus suggesting that BRD4 inhibitors influenced PRV attachment (Fig 5A). Similar phenomena were also observed in VSV and PRRSV attachment (Fig 5B and 5C). To rule out the possibility that BRD4 inhibitors might influence the viral envelope and inhibit viral attachment, we pre-treated PRV with BRD4 inhibitors at 37°C for 2 h and performed dialysis to remove the compounds. RT-qPCR analysis indicated that treatment of PRV with DMSO, JQ-1, OTX-015 and I-BET 151 did not alter viral attachment (S3A Fig). Immunoblotting analysis of PRV glycoprotein E (gE) indicated that BRD4 inhibitors decreased PRV attachment to cells (Fig 5D), a finding verified by immunofluorescence analysis (S3B Fig). Furthermore, we labeled the genomes of PRV and ECTV with 5-ethynyl-2'-deoxyuridine (EdU) and examined viral attachment with Apollo staining. When cells were treated with JQ-1, OTX-015 and I-BET 151, the cell fluorescence intensity of either PRV or ECTV was significantly weaker than that of control cells or cells treated with DMSO (Fig 5E and 5F).

In addition, we rescued BRD4 expression in BRD4-depleted cells by transfection of a plasmid for BRD4 expression that was resistant to both BRD4 shRNAs (Fig 5G). As with BRD4 inhibition, knockdown of BRD4 decreased the attachment of PRV and VSV to host cells, and exogenous expression of BRD4 brought the level of viral attachment to that on Scramble cells (Fig 5H and 5I). These results demonstrate that BRD4 inhibition attenuates viral attachment.

### BRD4 inhibition activates antiviral innate immunity

**Fig 6:**
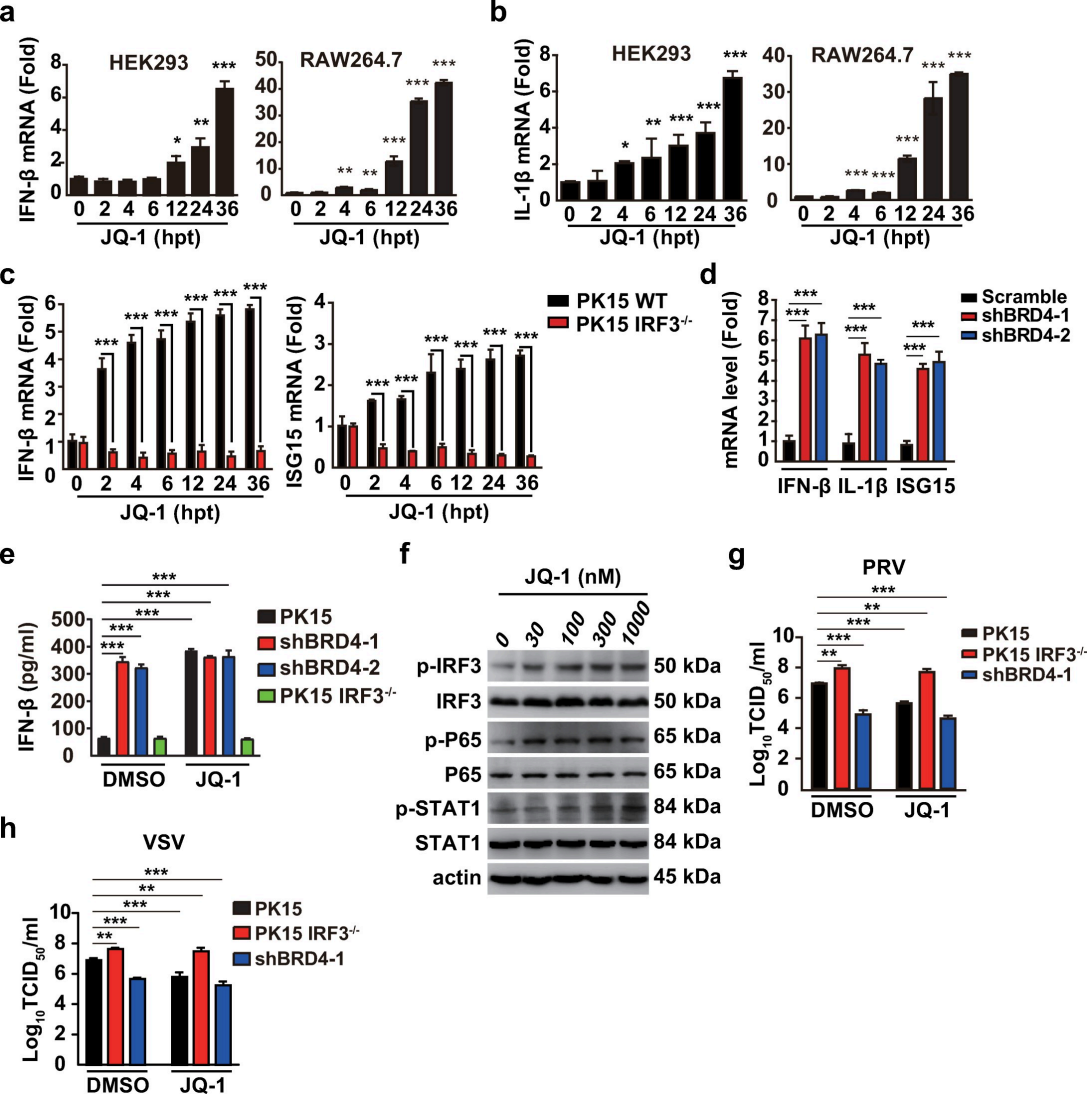
BRD4 inhibition activates antiviral innate immune responses. (a and b) IFN-β (a) and IL-1β (b) mRNA was assessed with RT-qPCR analysis in HEK293 and RAW264.7 cells treated with JQ-1 (1 μM) at 0, 2, 4, 6, 12, 24 and 36 hpt. (c) IFN-β and ISG15 mRNA were assessed with RT-qPCR analysis in PK15 and IRF^−/−^cells treated as in a. (d) IFN-β, IL-1β and ISG15 mRNA were assessed with RT-qPCR analysis in Scramble, shBRD4-1 and shBRD4-2 PK15 cells. (e) IFN-β was quantified by ELISA in PK15, IRF3^−/−^, shBRD4-1 and shBRD4-2 cells treated with DMSO or JQ-1 (1 μM) for 24 h. (f) Phospho-IRF3, phospho-P65, phospho-STAT1, total IRF3, P65 and STAT1 were assessed with immunoblotting analysis in HEK293 cells treated with DMSO and JQ-1 (0–1000 nM) for 24 h. Actin served as a loading control. (g and h) Viral titer was assessed with TCID_50_ assays in PK15, IRF3^−/−^and shBRD4-1 cells infected with PRV-QXX (g, MOI = 0.1) or VSV-GFP (h, MOI = 0.001) and treated with DMSO or JQ-1 (1 μM) for 24 h. All data are shown as mean ± SD based on three independent experiments. * P < 0.05, ** P < 0.01, *** P < 0.001 determined by two-tailed Student’s *t*-test.

Because host innate immunity defends against infection with both DNA and RNA viruses, we investigated the role of BRD4 inhibition in innate immune activation. We assessed the expression of IFN-β and the proinflammatory cytokine interleukin-1β (IL-1β) in HEK293 and RAW264.7 cells treated with JQ-1 for 0–36 h. By RT-qPCR analysis, we found that both IFN-β and IL-1β mRNAs were stimulated by JQ-1 in a time-dependent manner (Fig 6A and 6B). Because IRF3 contributes to IFN-β induction, we examined IFN-β expression in IRF3^-/-^ PK15 cells to determine whether the JQ-1 triggered IFN-β expression was dependent on IRF3. RT-qPCR analysis indicated that although JQ-1 induced IFN-β expression in PK15 cells, IFN-β mRNA in IRF3^-/-^ cells did not respond to JQ-1 treatment (Fig 6C). We also detected the expression of IFN-stimulated gene 15 (ISG15) and found that the pattern of ISG15 expression in PK15 and IRF3^-/-^ cells was similar to that of IFN-β (Fig 6C). We further assessed the expression of IFN-β, IL-1β and ISG15 in PK15 cells with BRD4 knockdown. In agreement with the results after JQ-1 treatment, BRD4 knockdown enhanced IFN-β, IL-1β and ISG15 mRNA expression (Fig 6D). Furthermore, IFN-β secretion in the culture medium of shBRD4-1 and shBRD4-2 PK15 cells was significantly greater than that in the culture medium of PK15 and IRF3^−/−^ cells, as assessed with ELISA (Fig 6E). After JQ-1 treatment, compared with DMSO treatment, PK15 cells generated more IFN-β (Fig 6E). The IFN-β secretion from IRF3^−/−^, shBRD4-1 and shBRD4-2 PK15 cells treated with JQ-1 was approximately equal to that from cells treated with DMSO (Fig 6E). We treated HEK293 cells with JQ-1 and further assessed innate immune activation with immunoblotting analysis. As shown in Fig 6F, phosphorylation of IRF3, P65 and STAT1 was up-regulated when cells were challenged with JQ-1 for 24 h, thus suggesting that innate immunity and IFN signaling were activated.

Next, we detected the infection of PRV and VSV in PK15, IRF3^−/−^ and shBRD4-1 cells in the presence of DMSO or JQ-1. IRF3 knockout promoted, but BRD4 knockdown inhibited, PRV and VSV infection in cells treated with DMSO (Fig 6G and 6H). After JQ-1 treatment, PRV and VSV infection were decreased in only PK15 cells (Fig 6G and 6H). JQ-1 treatment had no additional effects on PRV and VSV infection in IRF3^−/−^and shBRD4-1 PK15 cells (Fig 6G and 6H). Together, these data demonstrate that BRD4 inhibition boosts antiviral innate immunity.

### BRD4 inhibition leads to DDR

**Fig 7:**
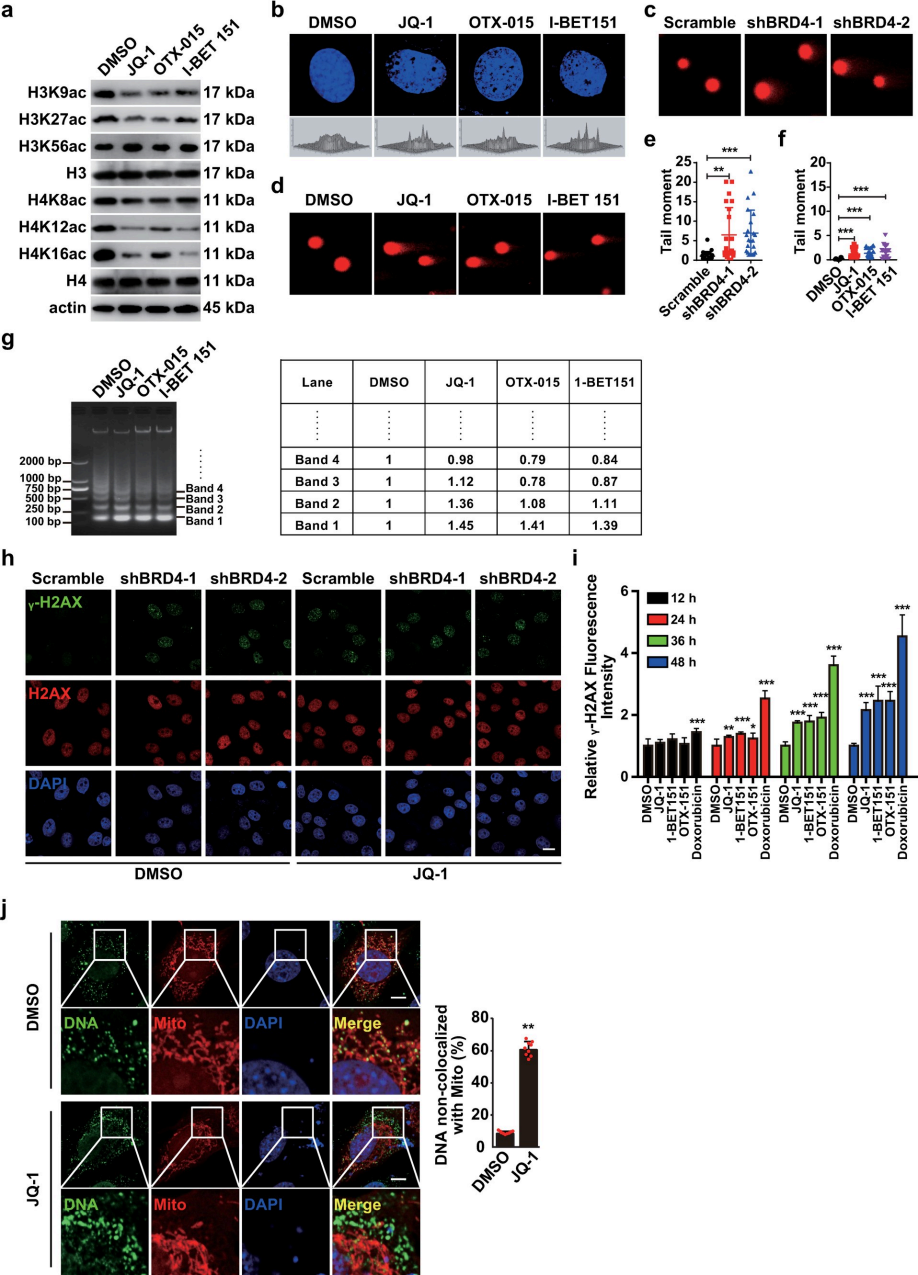
BRD4 inhibition induces chromatin decompaction and DDR. (a) Different acetylated lysine residues of histone H3 and H4 were assessed with immunoblotting in HEK293 cells treated with DMSO, JQ-1 (1 μM), OTX-015 (10 μM) and I-BET 151 (10 μM) at 37°C for 24 h. Actin served as a loading control. (b) Nuclear DNA structure was assessed with DAPI staining in NIH/3T3 cells treated with DMSO, JQ-1 (1 μM), OTX-015 (10 μM) and I-BET 151 (10 μM) for 24 h. (c and d) DNA damage was detected with comet assays in Scramble, shBRD4-1 and shBRD4-2 PK15 cells (c) or in NIH/3T3 cells (d) treated with DMSO, JQ-1 (1 μM), OTX-015 (10 μM) and I-BET 151 (10 μM) for 24 h. (e and f) Quantification of the tail moment for c (e) and d (f). Data are shown as mean ± SD of the tail moment of n = 22 cells. ** P < 0.01, *** P < 0.001 determined by two-tailed Student’s *t-*test. (g) Chromatin compaction was assessed with micrococcal nuclease assays in NIH/3T3 cells treated with DMSO, JQ-1 (1 μM), OTX-015 (10 μM) and I-BET 151 (10 μM) for 24 h. Quantification of band intensity by densitometry is shown on the right. (h) **γ**-H2AX and H2AX were assessed with immunofluorescence in Scramble, shBRD4-1 and shBRD4-2 PK15 cells treated with DMSO or JQ-1 (1 μM) for 24 h. Scale bar, 10 μm. (i) Quantification of **γ**-H2AX signal in NIH/3T3 cells treated with DMSO, JQ-1 (1 μM), OTX-015 (10 μM), I-BET 151 (10 μM) and doxorubicin (100 nM) for 12–48 h by Cytation5 (Biotek). Data are shown as mean ± SD based on three independent experiments. * P < 0.05, ** P < 0.01, *** P < 0.001 determined by two-tailed Student’s *t*-test. (j) Co-localization of mitochondria with cytosolic DNA was assessed with immunofluorescence with antibodies against Tom20 (Mito) and dsDNA (DNA) in NIH/3T3 cells treated with DMSO and JQ-1 (1 μM) for 24 h. Quantification of DNA non-colocalized with mitochondria is shown on the right. Data are shown as mean ± SD of the tail moment of n = 22 cells. ** P < 0.01 determined by two-tailed Student’s *t-*test. Scale bar, 10 μm.

BRD4 is involved in maintaining chromatin structure [12], we investigated whether BRD4 inhibition might induce the DDR to activate innate immunity. Because BRD4 is a histone acetyltransferase [16], we assessed whether BRD4 inhibition might induce changes in genome structure. Treatment of HEK293 cells with JQ-1, OTX-015 and I-BET 151 markedly decreased H3K9, H3K27, H4K8, H4K12 and H4K16 acetylation, but did not influence H3K56 acetylation, in agreement with previous findings [16] (Fig 7A). Treatment with JQ-1, OTX-015 and I-BET 151, compared with DMSO, resulted in chromatin decompaction in NIH/3T3 cells, as indicated by DAPI staining (Fig 7B). Comet assays revealed that DNA was broken in PK15 cells with BRD4 knockdown or in NIH/3T3 cells treated with BRD4 inhibitors (Fig 7C–7F). In addition, BRD4 inhibitors increased digestion by micrococcal nuclease, thus demonstrating a more ‘open’ overall chromatin structure (Fig 7G).

Because depletion of BRD8 induces the DDR in non-stressed cells [40], we sought to test whether BRD4 inhibition might induce the DDR. DNA double-stranded breaks can rapidly result in the phosphorylation of H2AX at serine 139 (γ-H2AX), which is the most sensitive marker that can be used to examine the DDR [41]. Therefore, we assessed γ-H2AX through immunofluorescence analysis to examine whether BRD4 inhibition induced the DDR. As shown in Fig 7H, γ-H2AX was observed in JQ-1-treated control cells and in cells with BRD4 knockdown. Quantification of γ-H2AX signals in NIH/3T3 cells showed time-dependent effects of BRD4 inhibitors and doxorubicin on the DDR (Fig 7I). Moreover, we detected micronuclei-like cytoplasmic dsDNA in NIH/3T3 cells treated with JQ-1, and most of the dsDNA was not colocalized with mitochondria (Fig 7J). These data suggest that BRD4 inhibition induces the DDR.

### BRD4 inhibition leads to DDR-dependent cGAS activation

**Fig 8:**
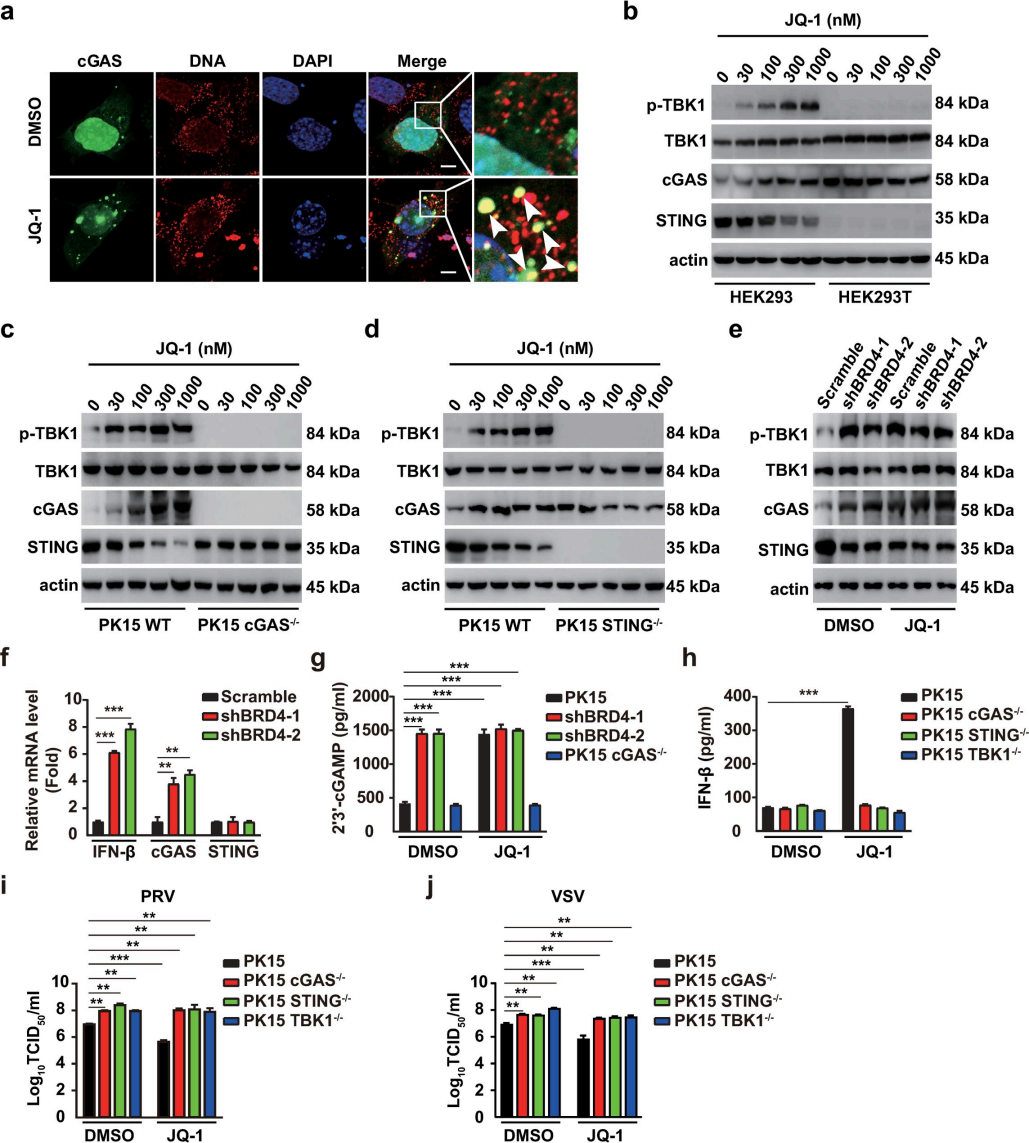
BRD4 inhibition stimulates DDR-dependent cGAS activation. (a) Co-localization of cGAS with cytosolic DNA was assessed with immunofluorescence in cGAS-EGFP transfected NIH/3T3 cells treated with DMSO and JQ-1 (1 μM) for 24 h. Scale bar, 10 μm. (b) Phospho-TBK1, total TBK1, cGAS and STING were assessed with immunoblotting analysis in HEK293 and HEK293T cells treated with JQ-1 (0–1000 nM) for 24 h. Actin served as a loading control. (c and d) Phospho-TBK1, total TBK1, cGAS and STING were assessed with immunoblotting analysis in PK15 WT (c and d), PK15 cGAS^-/-^(c) and PK15 STING^-/-^ (d) cells treated with JQ-1 (0–1000 nM) for 24 h. Actin served as a loading control. (e) Phospho-TBK1, total TBK1, cGAS and STING were assessed with immunoblotting analysis in Scramble, shBRD4-1 and shBRD4-1 PK15 cells treated with DMSO and JQ-1 (1 μM) for 24 h. Actin served as a loading control. (f) IFN-β, cGAS and STING mRNAs were assessed with RT-qPCR analysis in Scramble, shBRD4-1 and shBRD4-1 PK15 cells. Data are shown as mean ± SD based on three independent experiments. ** P < 0.01, *** P < 0.001 determined by two-tailed Student’s *t*-test. (g) 2′3′-cGAMP was quantified by ELISA in PK15, shBRD4-1, shBRD4-2 and PK15 cGAS^−/−^ cells treated with DMSO or JQ-1 (1 μM) for 24 h. Data are shown as mean ± SD based on three independent experiments. *** P < 0.001 determined by two-tailed Student’s *t*-test. (h) IFN-β was quantified by ELISA in PK15, PK15 cGAS^-/-^, PK15 STING^−/−^ and PK15 TBK1^−/−^ cells treated with DMSO and JQ-1 (1 μM) for 24 h. Data are shown as mean ± SD based on three independent experiments. *** P < 0.001 determined by two-tailed Student’s *t*-test. (i and j) Viral titer was assessed with TCID_50_ assays in PK15, PK15 cGAS^−/−^, PK15 STING^−/−^ and PK15 TBK1^−/−^ cells infected with PRV-QXX (i, MOI = 0.1) and VSV-GFP (j, MOI = 0.001) and treated with DMSO or JQ-1 (1 μM) for 24 h. Data are shown as mean ± SD based on three independent experiments. ** P < 0.01, *** P < 0.001 determined by two-tailed Student’s *t*-test.

Because cGAS is a predominant and general sensor of cytosolic DNA [21], we examined whether cGAS might be involved in JQ-1-induced innate immune activation. cGAS activation requires binding to dsDNA [22], by first determining the co-localization of cGAS and dsDNA. We observed that JQ-1 treatment induced condensed the structure of cGAS, some of which co-localized with cytosolic dsDNA, thus suggesting potential cGAS activation (Fig 8A). Furthermore, through RT-qPCR analysis in PK15 cells with ablation of cGAS, STING and TBK1, we found that JQ-1-triggered IFN-β and IL-1β expression was abolished (S4A and S4B Fig). We next analyzed the activation of the cGAS/STING/TBK1 axis through immunoblotting. As shown in Fig 8B, JQ-1 treatment induced phosphorylation of TBK1 in HEK293 cells. cGAS expression was up-regulated by JQ-1 treatment, possibly because cGAS is an ISG (Fig 8B) [42]. STING expression was decreased by JQ-1 treatment, possibly because STING can be degraded through an autophagy pathway [43]. A similar phenomenon was also observed in PK15 cells (Fig 8C and 8D). In contrast, HEK293T cells, which lack STING [44], exhibited no phosphorylation of TBK1 in response to JQ-1 treatment (Fig 8B). Ablation of cGAS and STING also abolished JQ-1 induced phosphorylation of TBK1 in PK15 cells (Fig 8C and 8D). BRD4 silencing enhanced phosphorylation of TBK1 and cGAS expression and perturbed STING expression (Fig 8E). cGAS mRNA, but not STING mRNA, was up-regulated in cells with BRD4 knockdown (Fig 8F).

ELISA of 2′3′-cGAMP, the enzymatic product of cGAS, indicated that BRD4 knockdown enhanced 2′3′-cGAMP production (Fig 8G). JQ-1 treatment induced 2′3′-cGAMP production in PK15 cells but not in cGAS^−/−^ PK15 cells, thus indicating that BRD4 inhibition activated cGAS (Fig 8G). IFN-β secretion was abrogated in cGAS^-/-^, STING^-/-^ and TBK1^-/-^ PK15 cells treated with JQ-1 (Fig 8H). Finally, we investigated the role of the cGAS/STING/TBK1 axis in JQ-1-induced antiviral activity. Viral titration indicated that ablation of cGAS, STING and TBK1 enhanced PRV and VSV infection in cells treated either with DMSO or with JQ-1, thus suggesting that the cGAS-mediated innate immune pathway was required for the antiviral role of JQ-1 (Fig 8I and 8J). These results indicate that BRD4 inhibition leads to the activation of cGAS-dependent innate immune responses.

### JQ-1 inhibits viral infection *in vivo*

**Fig 9:**
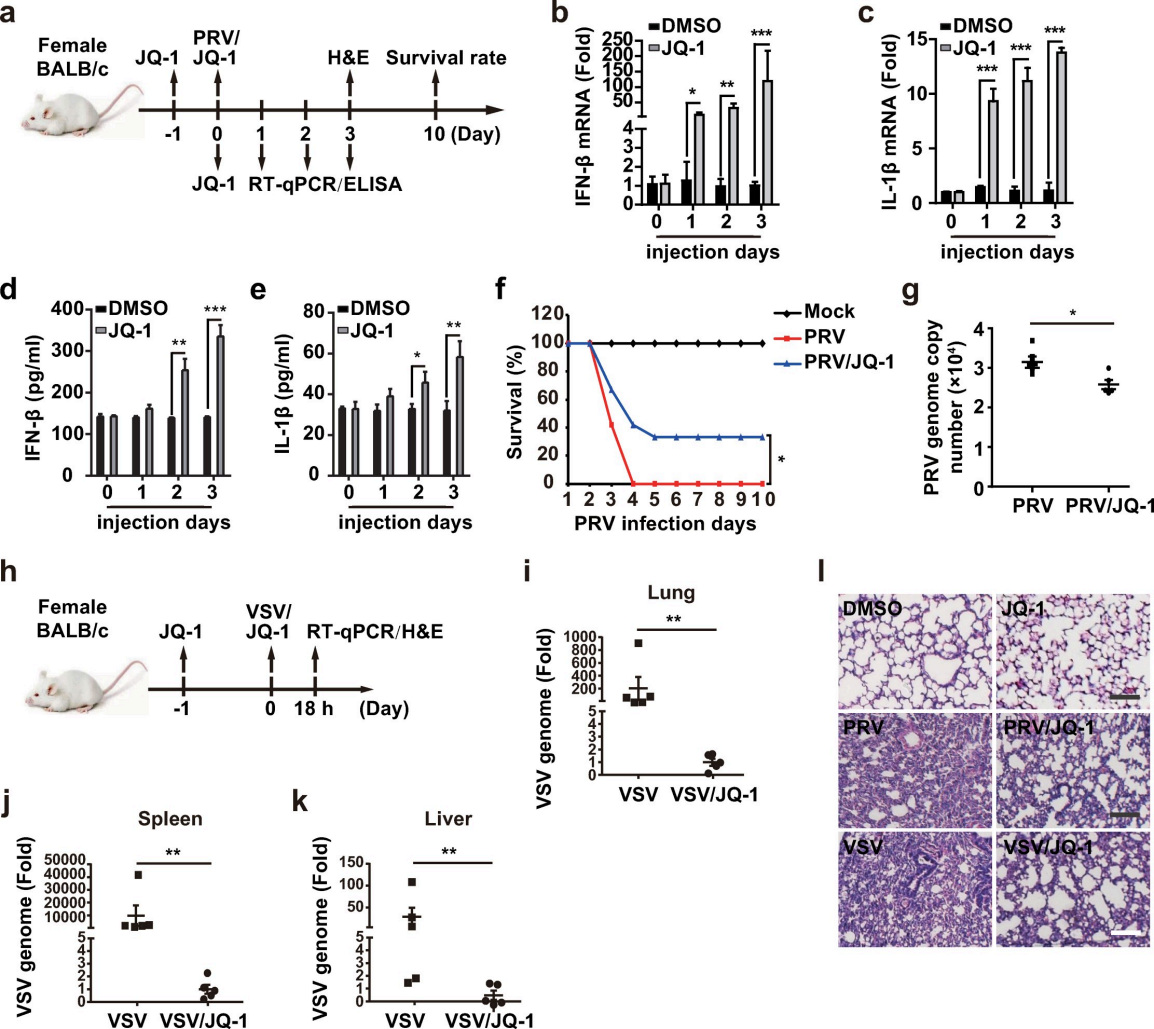
JQ-1 restricts viral infection *in vivo*. (a) Experimental strategy for PRV-QXX challenge and JQ-1 treatment in mice. (b and c) IFN-β (b) and IL-1β (c) mRNA were assessed with RT-qPCR analysis in the lungs of mice (n = 4 per group). (d and e) IFN-β (d) and IL-1β (e) were quantified by ELISA in the serum in mice (n = 5 per group). (f) The survival rate was monitored daily for 10 days after PRV and PRV/JQ-1 challenge. (n = 12 per group). (g) The PRV genome was assessed with RT-qPCR analysis in the lungs of mice at 3 days post treatment of PRV and PRV/JQ-1 (n = 5 per group). (h) Experimental strategy for VSV-GFP challenge and JQ-1 treatment in mice. (i-k) The VSV genome was assessed with RT-qPCR analysis in the lung (i), spleen (j) and liver (k) of mice at 18 h post treatment of VSV and VSV/JQ-1 (n = 5 per group). (l) H&E stained images of lungs sections from mice treated as in g and i. Scale bar, 100 μM. All data are shown as mean ± SD based on three independent experiments. * P < 0.05, ** P < 0.01, *** P < 0.001 determined by two-tailed Student’s *t*-test.

On the basis of the above findings, we examined the protective role of JQ-1 against PRV and VSV infection *in vivo*. Mice were intraperitoneally injected with JQ-1 for 0–3 days (Fig 9A). In agreement with our *in vitro* data, the expression of IFN-β and IL-1β mRNA in mouse lungs was significantly enhanced by JQ-1 injection (Fig 9B and 9C). The levels of IFN-β and IL-1β were up-regulated in the serum in mice injected from 2 day post JQ-1injection (Fig 9D and 9E). These data indicated that BRD4 inhibition activated the innate immune response *in vivo*. We then challenged mice with PRV-QXX and examined the protective role of JQ-1. We found that the mortality of mice injected with JQ-1 was significantly lower than that of mice injected with DMSO (Fig 9F). The PRV load was much higher in the lungs in control mice than JQ-1-injected mice, on the basis of RT-qPCR analysis of the viral genome (Fig 9G). Furthermore, we detected the inhibitory effect of JQ-1 on VSV-GFP infection *in vivo* (Fig 9H). The replication of the VSV genome in mouse lung, spleen and liver was markedly inhibited in response to JQ-1 treatment (Fig 9I–9K). Lung injury caused by PRV and VSV infection was greatly attenuated upon JQ-1 treatment, because less infiltration of inflammatory cells was detected in the lungs in JQ-1-treated mice than DMSO-treated mice (Fig 9L). These results revealed that BRD4 inhibition exerts antiviral activity *in vivo*.

## Discussion

**Fig 10:**
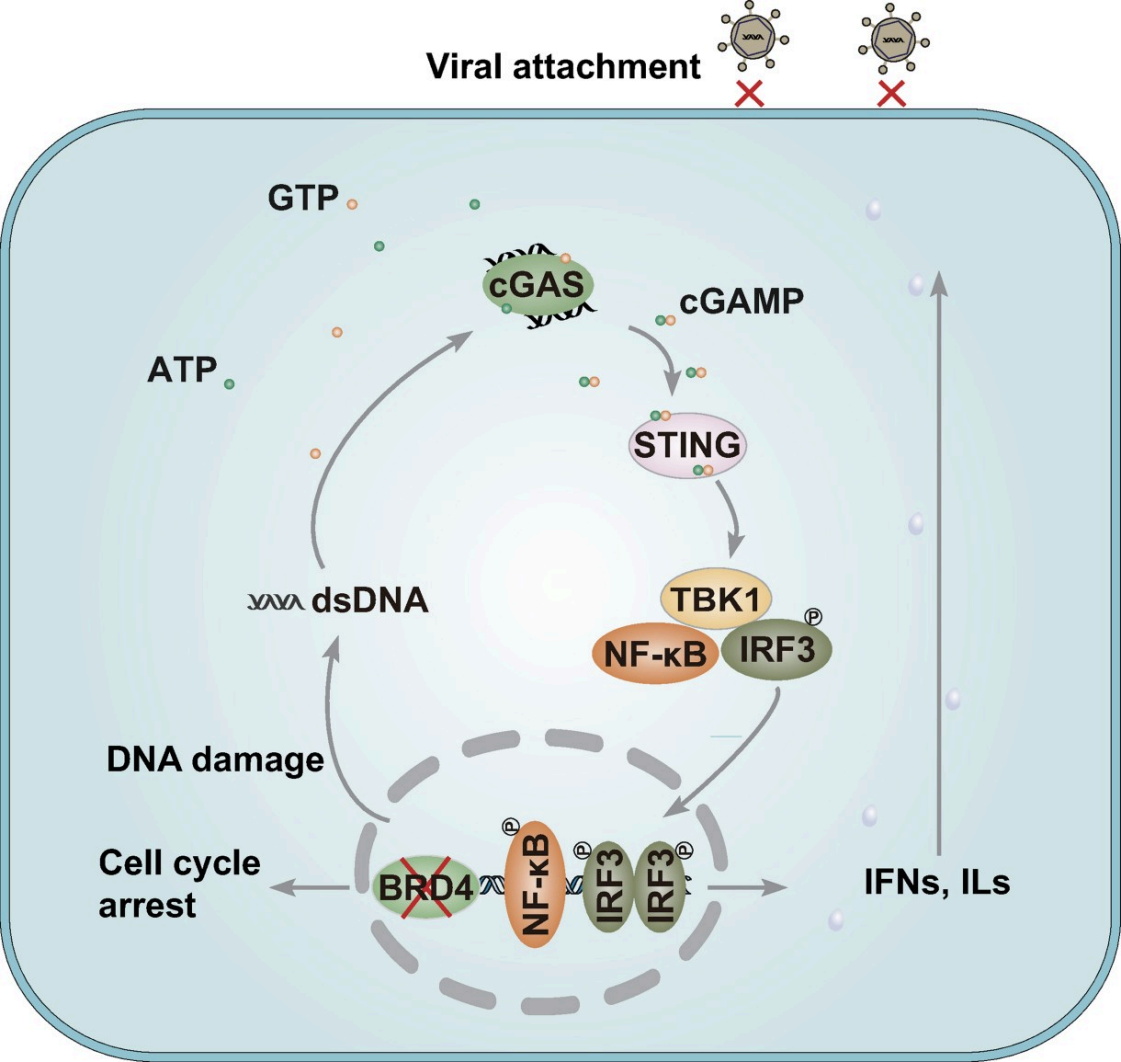
A schematic model showing BRD4 inhibition in antiviral innate immunity. BRD4 inhibition leads to DDR-induced activation of cGAS-mediated innate immune pathway, which exhibits broadly antiviral activity through impairment of viral attachment.

BRD4 is a master regulator of gene expression that plays important roles in recognizing and modifying histone acetylation. However, the effects of BRD4 on the maintenance of global chromatin structure vary in different contexts, and whether BRD4 is involved in antiviral innate immunity is elusive. Here, we report that BRD4 inhibition induces cellular genomic instability and increases in the levels of cytoplasmic DNA, which in turn promotes the secretion of type I interferon and proinflammatory cytokines through cGAS/STING/TBK1/IRF3 signaling, thus exhibiting broad antiviral activity through attenuation of viral attachment (Fig 10). Beyond cGAS, a number of cytoplasmic DNA sensors have been identified, such as DNA-dependent activator of IFN-regulatory factor and DEAD box polypeptide 41 [45]. Because cGAS is the predominant and general sensor of cytosolic DNA [21], and cGAS knockout blocked the antiviral effectors in response to BRD4 inhibition (Fig 8C–8H), other DNA sensors did not play a significant role in our experimental context.

Although BRD4-mediated regulation of chromatin structure is likely to be complex and context-specific [16–19], we provide evidence that BRD4 inhibition induces chromatin decompaction, a finding similar to previous observations [17, 18]. DAPI staining and micrococcal nuclease assays suggested that treatment of cells with BRD4 inhibitors caused chromatin decompaction and fragility (Fig 7B–7G). Bowry et al. have indicated that BET inhibitors activate RAD51, thus slowing replication forks and suppressing DNA damage [46]. However, other researchers have indicated that BRD4 inhibition decreases RAD51 expression and homologous recombination repair, thus potentially inducing DNA damage in prostate and breast cancer cells [47, 48]. Nevertheless, we demonstrate that BRD4 inhibition induces the DDR. First, DNA comet assays showed that DNA injury occurred in cells with BRD4 knockdown or BRD4 inhibitor treatment (Fig 7C–7F). Second, immunofluorescence analysis of γ-H2AX demonstrated that BRD4 inhibition resulted in double-strand DNA breaks (Fig 7H and 7I).

Although BRD4 inhibition has been proposed to induce apoptosis in cholangiocarcinoma cells [49], we did not detect significant apoptosis in cells in response to BRD4 inhibition. One possible explanation is that BRD4-inhibition induced DDR to a lesser extent than the chemotherapeutic agent doxorubicin [50, 51] and thus did not substantially promote apoptosis. Another possible reason is that P53 is not activated under treatment with BRD4 inhibitors (S1C Fig). Consistent with this possibility, JQ-1 treatment has been found not to activate P53 and P21 in primary pediatric B-precursor acute lymphoblastic leukemia cells [52]. The discrepancy in BRD4 inhibition in apoptosis and DNA repair in different cell types must be further clarified.

Previous studies have demonstrated that BRD4 facilitates viral infection through regulation of viral gene transcription [53, 54]. Intriguingly, we demonstrated that BRD4 inhibition attenuates attachment of a range of viruses (Fig 5). IFNs induce the expression of ISGs through the IFN-α/β receptor, Janus kinase-signal transducer and activator of transcription signaling pathway. In each cell, 500 to 1,000 ISGs are induced and subsequently block multiple stages of the viral life cycle [55, 56]. Our previous study has demonstrated that porcine cholesterol 25-hydroxylase (CH25H) is regulated by IFNs, and 25-hydroxycholesterol (25HC) inhibits PRV entry [57]. CH25H expression is also induced in response to infection with Zika virus, Singapore grouper iridovirus and red-spotted grouper nervous necrosis virus, and subsequently blocks viral attachment by 25HC [58, 59]. Moreover, the IFN-induced transmembrane proteins (IFITM) located on the plasma membrane and endosomes have been reported to exhibit broad-spectrum antiviral activity [60, 61]. IFITM proteins may modulate the fluidity of cellular membranes and interfere with a step in viral replication preceding fusion of the viral and cellular membranes [62]. Because BRD4 inhibition activates IFN-β expression (Fig 6A–6E), we postulate that CH25H or IFITM proteins may be up-regulated to inhibit viral attachment when BRD4 is inhibited. Of course, other ISGs may participate in BRD4 inhibition-induced antiviral activity.

Several studies have suggested that BRD4 positively regulates inflammatory gene expression. I-BET inhibits the expression of key inflammatory genes in activated macrophages and primary human monocytes [63, 64]. Deletion of BRD4 promotes IκBα transcription and influences LPS-induced inflammatory gene expression and cytokine production in bone marrow-derived macrophages [65]. In addition, BRD4 binds the IFN-β promoter and facilitates IFN-β expression [66]. However, we found that BRD4 inhibition triggers the innate immune response through the cGAS-STING pathway (Fig 8). Harnessing innate immunity is emerging as a new possibility for cancer therapy [67]. The intrinsic relevance of STING-associated signaling in tumorigenesis has been a recent concern of oncologists, and STING agonists have been tested in early clinical trials [68, 69]. Moreover, BRD4 is largely acknowledged for its roles in organization of super-enhancers and oncogene expression in cancer [70, 71]. We suggest another beneficial effect of BRD4 inhibitors in cancer immunotherapy.

## Materials and methods

### Ethics statement

Experiments involving animals were approved by the Committee on the Ethics of Animal Care and Use of National Research Center for Veterinary Medicine (Permit 20160313091). The study was conducted in accordance with the Guide for the Care and Use of Animals in Research of the People's Republic of China.

### Mice

Female 6–8-week-old BALB/c mice were purchased from the Center of Experimental Animal of Zhengzhou University (Zhengzhou, China) and maintained in a specific pathogen–free animal facility according to the guide for the care and use of laboratory animals and the related ethical regulations instilled at Henan Agricultural University.

Mice were intraperitoneally injected with JQ-1 (50 mg/kg) for 1 day. On the next day, mice were intraperitoneally injected with JQ-1 (50 mg/kg) again. After 1 h, mice were intranasally infected with PRV-QXX (5×10^3^ TCID_50_ per mouse) for 0–10 days or with VSV-GFP (5×10^7^ pfu per gram body weight) for 18 h.

### Cells and viruses

Pig kidney (PK-15) cells (CCL-33, ATCC), human cervical cancer (HeLa) cells (CL-82, ATCC), human embryonic kidney (HEK293) cells (CRL-1573, ATCC), HEK293 cells transformed with SV40 large T antigen (HEK293T) (CRL-11268, ATCC), mouse fibroblast (NIH/3T3) cells (CRL-1658, ATCC), African green monkey kidney (Vero) cells (CL-81, ATCC), human lung carcinoma (A549) cells (CCL-185, ATCC), mouse macrophages (RAW264.7) (TIB-71, ATCC) and canine kidney (MDCK) cells (CCL-34, ATCC) were grown in monolayers at 37°C under 5% CO_2_.

All cells were cultured in DMEM (10566–016, GIBCO) supplemented with 10% FBS (10099141C, GIBCO), 100 units/mL penicillin and 100 μg/mL streptomycin sulfate (B540732, Sangon). African green monkey kidney (MARC-145) cells and cGAS^-/-^, STING^-/-^, TBK1^-/-^, IRF3^-/-^, IFNAR1^-/-^ PK15 cells were cultivated as previously described [72, 73].

PRV-GFP, PRV-QXX and PRRSV-BJ4 were used as previously described [57, 72, 74]. VSV-GFP, NDV-GFP and H1N1-PR8 were gifts from Yong-Tao Li (Henan Agricultural University, China) [75]. HSV-F was a gift from Chun-Fu Zheng (Fujian Medical University, China) [76]. ECTV was a gift from Han-Zhong Wang (Wuhan Institute of Virology, Chinese Academy of Sciences) [77].

### Chemical reagents

ML324 (HY-12725), A196 (HY-100201), Irinotecan (HY-16562), Doxorubicin (HY-15142), EPZ-6438 (HY-13803), GSK503 (HY-12856), 5-Azacytidine (HY-10586), JQ-1 (HY-13030), OTX-015 (HY-15743), I-BET151 (HY-13235) and wortmannin (HY-10197) were from MedChemExpress. Lipofectamine 3000 (L3000015), CellMask Green (C37608) and CellMask Deep Red (C10046) Plasma Membrane Stain were from Invitrogen.

### Antibodies

The antibodies anti-H3K9ac (#2594), anti-H3K27ac (#8173), anti-H3K56ac (#07-677-1S), anti-H4K8ac (#2594), anti-H4K12ac (#13944), anti-H4K16ac (#13534), anti-H3 (#4499), anti-H4 (#13919), anti-BRD4 (#13440), anti-Tom20 (#42406), anti-γ-H2AX (#80312), anti-H2AX (#7631), anti-p-TBK1(Cell #5483), anti-TBK1 (#3504), anti-p-IRF3 (#29047y), anti-IRF3 (#11904), anti-p-P65 (#3033), anti-P65 (#8242), anti-p-STAT1(#9167), anti-STAT1(#9172), anti-STING (#3337) and anti-p-P53 (#9286) were from Cell Signaling Technology; anti-cGAS (26416–1-AP), anti-P53 (10442-1-AP), anti-MDM2 (19058-1-AP) and anti-P21 (#10355-1-AP) were from Proteintech; anti-dsDNA (MAB1293) was from Millipore; and anti-actin (A1978) was from Sigma. Antiserum against PRV gE was generated by immunization of mice with purified recombinant gE.

### Plasmids

The plasmid for expression of GFP-tagged cGAS was used as previously described [39]. The plasmid for expression of flag-tagged BRD4 that was resistant to BRD4 shRNAs was synthesized and constructed by GenScript.

### Cell viability and proliferation assays

Cell viability was evaluated with Cell Counting Kit-8 (CCK-8) assays, according to the manufacturer’s instructions (GK3607, DingGuo, Beijing). For cell viability assays, cells were seeded at 1 × 10^4^ per well into 96-well plates. On the next day, the medium was changed to DMEM/10% FBS supplemented with different concentrations of JQ-1, OTX-015 and I-BET151 for 48 h. CCK-8 (10 μl) was then added to each well, and the cells were incubated for 3 h at 37°C. The absorbance was detected at 450 nm with a microplate reader (VARIOSKAN FLASH, ThermoFisher).

For cell proliferation assays, PK15, Scramble, shBRD4-1 and shBRD4-2 cells were seeded at 1 × 10^4^ per well into 96-well plates. On the next day, the medium was changed to DMEM/10% FBS, and cells were cultured for the indicated times. CCK-8 (10 μl) was then added to each well, and the cells were incubated for 3 h at 37°C. The absorbance was detected at 450 nm with a microplate reader (ThermoFisher).

### Flow cytometry assay

For GFP reporter assays, PK15 cells were pre-treated with compound for 4 h, and then infected with PRV-GFP (MOI = 0.01) and simultaneously treated with compounds for 36 h. Cells were digested with trypsin-EDTA (25200072, GIBCO), collected by centrifugation and suspended in phosphate-buffered saline (PBS). The percentage of GFP positive cells was measured by flow cytometry on a CytoFLEX instrument.

For apoptosis assays, HeLa and HEK293 cells were seeded at 1.2 × 10^5^ per well into 24-well plates. On the next day, the medium was changed to DMEM/10% FBS supplemented with DMSO, JQ-1 (1 μM), OTX-015 (10 μM) and I-BET151 (10 μM) for 24 h. Annexin V/PI staining was performed with a Dead Cell Apoptosis Kit with Annexin V FITC and PI (V13242, ThermoFisher) according to the manufacturer’s instructions. The percentage of dead cells (positive for both Annexin V and PI) was measured by flow cytometry on a CytoFLEX instrument (BECKMAN COULTER).

For cell cycle analysis, PK15 and HEK293 cells were seeded at 1.2×10^5^ per well into 24-well plates. On the next day, the medium was changed to DMEM/10% FBS supplemented with DMSO, JQ-1 (1 μM), OTX-015 (10 μM) and I-BET151 (10 μM) for 1, 4, 8, 12 and 24 h. Cells were then digested with trypsin-EDTA and resuspended in PBS containing 5 μg/ml Hoechst 33342 (561908, BD) at a concentration of 1 × 10^6^ cells/ml. After incubation for 1 h at 37°C, cell cycle profiles were collected by flow cytometry on a CytoFLEX instrument. All data were analyzed in FlowJo software.

### Immunoblotting analysis

Cells were collected in lysis buffer (50 mM Tris-HCl, pH 8.0, 150 mM NaCl, 1% Triton X-100, 1% sodium deoxycholate, 0.1% SDS and 2 mM MgCl_2_) supplemented with protease and phosphatase inhibitor cocktail (HY-K0010 and HY-K0022, MedChemExpress). The protein concentrations of the lysates were quantified with a BCA Protein Assay Kit (BCA01, DINGGUO Biotechnology). Protein samples were separated by SDS-PAGE and transferred to transfer membranes (ISEQ00010, Millipore), which were incubated in 5% nonfat milk (A600669, Sangon) at room temperature for 1 h. The membrane was incubated with the primary antibody at 4°C overnight and then incubated with horseradish-peroxidase-conjugated secondary antibody (709-035-149 or 715-035-150, Jackson ImmunoResearch Laboratories) for 1 h. Immunoblotting results were visualized with Luminata Crescendo Western HRP Substrate (WBLUR0500, Millipore) on a GE AI600 imaging system.

### Immunofluorescence

Cells grown on coverslips (12-545-80, ThermoFisher) were fixed with 4% paraformaldehyde for 30 min, permeabilized in 0.1% Triton X-100 and then incubated with PBS containing 10% FBS (10% FBS/PBS) with the primary antibody for 1 h at room temperature. After the cells were washed three times with PBS and then were labeled with 10% FBS/PBS containing the fluorescent secondary antibody (#A-11034, #A-11001, #A-11011 and #A-11004, ThermoFisher) for 1 h. The cells were finally washed in PBS and mounted in ProLong Diamond with DAPI (#P36971, Invitrogen). Images were captured on a Zeiss LSM 800 confocal microscope and processed in ImageJ software for quantitative image analysis.

### RT-qPCR

Total RNA was isolated with TRIzol Reagent (9108, TaKaRa) and subjected to cDNA synthesis with a PrimeScript RT reagent Kit (RR047A, TaKaRa). RT-qPCR was performed in triplicate by using SYBR Premix Ex Taq (RR820A, TaKaRa) according to the manufacturer’s instructions, and data were normalized to the level of β-actin expression in each individual sample. Melting curve analysis indicated formation of a single product in all cases. The 2^−ΔΔCt^ method was used to calculate relative expression changes.

For quantification of PRV genome copy numbers, the PCR product of PRV gH (187 bp) was cloned into the pGEM-T vector. A standard curve was prepared from serial 10-fold dilutions of the plasmid for the quantification of PRV genomic DNA. Primers used for RT-qPCR are as follows: porcine actin-Fw: 5′-CTGAACCCCAAAGCCAACCGT-3′, porcine actin-Rv: 5′-TTCTCCTTGATGTCCCGCACG-3′; porcine IFN-β-Fw: 5′- AGTTGCCTGGGACTCCTCAA-3′, porcine IFN-β-Rv: 5′- CCTCAGGGACCTCAAAGTTCAT-3′; porcine IL-1β-Fw: 5′- CCATCCACTGAGCCAGCCTT-3′, porcine IL-1β-Rv: 5′-TGCCAAGGACAGAGGACTGC-3′; porcine BRD4-Fw: 5′-CGCGATGCTCAGGAATTTGG-3′, porcine BRD4-Rv: 5′-TGCTGCTGTCACTGGATGAG-3′; porcine ISG15-Fw: 5′-ATGCCCCCTTGCCCTCTCCAGTG-3′, porcine ISG15-Rv: 5′-TCCGATGCCATCATGCAGTCCCT-3′; porcine c-Myc-Fw: 5′-CCACGAAACTTTGCCCACTG-3′, porcine c-Myc-Rv: 5′-TTCTCCTCCTCGTCGCAGTA-3′; porcine c-Jun-Fw: 5′-AGCAGAGCATGACCCTGAAC-3′, porcine c-Jun-Rv: 5′-ACTGGATTATCAGGCGCTCG-3′; human actin-Fw: 5′-GCACAGAGCCTCGCCTT-3′, human actin-Rv: 5′-CCTTGCACATGCCGGAG-3′; human IFN-β-Fw: 5′- CAGGAGAGCAATTTGGAGGA-3′, human IFN-β-Rv: 5′-CTTTCGAAGCCTTTGCTCTG-3′; human IL-1β-Fw: 5′- CCTGAAGCCCTTGCTGTAGT-3′, human IL-1β-Rv: 5′-AGCTGATGGCCCTAAACAGA-3′; mouse actin-Fw: 5′- CCCCATTGAACATGGCATTG-3′, mouse actin-Rv: 5′-ACGACCAGAGGCATACAGG-3′; mouse IFN-β-Fw: 5′- ATGAGTGGTGGTTGCAGGC-3′, mouse IFN-β- Rv: 5′- TGACCTTTCAAATGCAGTAGATTCA-3′; mouse IL-1β-Fw: 5′- GCAGAGCACAAGCCTGTCTTCC-3′, mouse IL-1β-Rv: 5′-ACCTGTCTTGGCCGAGGACTAAG-3′; PRV gH-Fw: 5′-CTCGCCATCGTCAGCAA-3′, PRV gH-Rv: 5′-GCTGCTCCTCCATGTCCTT-3′; PRRSV ORF7-Fw: 5′-AGATCATCGCCCAACAAAAC-3′, PRRSV ORF7-Rv: 5′-GACACAATTGCCGCTCACTA-3′; VSV N-Fw: 5′-GATAGTACCGGAGGATTGACGACTA-3′, VSV N-Rv: 5′- TCAAACCATCCGAGCCATTC-3′.

### RNA interference

Lentivirus-mediated gene silencing was conducted as previously described [72]. Briefly, shRNAs (Scramble: GCCACAACGTCTATATCATGG; shBRD4-1: GGAATGCTCAGGAATGTATCC; shBRD4-2: GGTACCAAACACAACACAAGC) were synthesized as double-strand oligonucleotides, cloned into the pLKO.1 vector (SHC001, Sigma) and cotransfected with packaging plasmids pMD2.G and psPAX2 (#12259 and #12260, Addgene) into HE293T cells. Lentiviruses were harvested at 48 h post-transfection and used to infect cells that were then selected with puromycin (4 μg/ml) for 7 days. Knockdown efficiency was determined by immunoblotting analysis.

### Comet assays

Cells were seeded in six-well plates and treated as described. Normal melting point agarose (NMA, 0.5%) was coated on frosted microscope slides. Approximately 10,000 cells in 10 μl DMEM were mixed with 75 μl low melting point agarose (LMA, 0.7%), and the mixture was dripped onto the precoated NMA layers. The third layers were prepared with 75 μl of 0.7% LMA. The cells were lysed in lysis buffer (2.5 M NaCl, 100 mM Na_2_EDTA, 10 mM Tris, pH 10.0, 1% Triton X-100 and 10% DMSO) for 2 h at 4°C. After lysis, the slides were placed in electrophoresis solution (300 mM NaOH, 1 mM Na_2_EDTA, pH >13) for 40 min, subjected to electrophoresis at 20 V (∼300 mA) for 25 min and subsequently neutralized with 0.4 mM Tris–HCl (pH 7.5). Finally, the cells were stained with PI (5 μg/mL) and evaluated on a Zeiss LSM 800 confocal microscope. DNA damage was measured in terms of tail moment in cometscore software.

### Micrococcal nuclease assays

PK-15 cells (5 × 10^6^) were homogenized in 500 μl of buffer A (10 mM HEPES, pH 7.4, 10 mM KCl, 1.5 mM MgCl_2_ and 250 mM sucrose), and the nuclei were isolated by centrifugation at 1000×g for 7 min. Then the nuclei were gently resuspended in 100 μl of nuclear buffer (50 mM Tris-HCl, pH 7.9, 5 mM CaCl_2_ and 100 μg/ml BSA) and incubated with 100 gel units of micrococcal nuclease (M0247S, New England BioLabs) at 37°C. After incubation for 10 min, 80 μl of micrococcal nuclease digestion buffer and 20 μl of stop buffer (250 mM EDTA, 5% SDS) were added, and this was followed by addition of 400 μl of lysis buffer (50 mM Tris-HCl, pH 7.6, 100 mM NaCl, 5 mM EDTA, 0.5% SDS, 1.5 mg/ml RNase) and incubation at 37°C for 30 min. The samples were treated with proteinase K (300 μg/ml) at 58°C for 2 h. After phenol-chloroform extraction, the genomic DNA was precipitated with 1/10 volume of 3 M sodium acetate (pH 5.2) and 2.5 volumes of ethanol. The air-dried DNA pellet was dissolved in 20 μl of ddH_2_O, and equal amounts- of DNA samples were separated on a 1.2% agarose gel. The results were visualized with ethidium bromide (A600195, Sangon) staining on a BIO-RAD Gel DOC^TM^ XR+ imaging system.

### Hemagglutination (HA) assays

NDV-GFP and H1N1-PR8 viruses from infected cells were harvested with two freeze–thaw cycles. The samples were then serially diluted with PBS in V-bottom 96-well plates and incubated with 0.5% of chicken red blood cells for 15 min at 37°C. The HA endpoint was the highest sample dilution at which agglutination was observed.

### Viral infection and viral titer assays

For viral infection, cells were incubated with virus for 1 h at 37°C at the indicated MOI. Then, the excess virus inoculum was removed by washing three times with PBS. Virus was harvested with two freeze–thaw cycles at the time point indicated.

For viral titer assays, cells were seeded in 96-well plates at a density of 1 × 10^4^ per well. On the next day, the cells were inoculated with serially diluted viruses (10^−1^–10^−12^ fold) for 1 h at 37°C. The excess virus inoculum was removed by washing three times with PBS. Then, 200 μl of maintenance medium (DMEM/2% FBS) was added to each well, and the cells were further cultured for 3–5 days. The cells demonstrating the expected cytopathic effect were observed daily, and the TCID_50_ value was calculated with the Reed-Muench method.

### ELISA

Cell culture supernatants were tested for IFN-β (ABCE-EL-P1819, Advanced BioChemicals) and 2′3′-cGAMP (501700, Caymanchem) with ELISA kits according to the manufacturer’s instructions.

Mouse serum was tested for IFN-β (42400, PBL assay science) and IL-1β (893829, R&D Systems) with ELISA kits according to the manufacturer’s instructions.

### EdU labeling and staining

For EdU labeling, cells were cultured in DMEM/10% FBS containing 25 μM EdU (C00031, RIBOBIO) and simultaneously infected with PRV-QXX (MOI = 1) or ECTV (MOI = 10) for 24 h. Edu-labeled viruses were harvested with two freeze–thaw cycles, and the virus titer was determined with TCID_50_ assays. For EdU staining, cells were incubated with Edu-labeled virus and CellMask Green or CellMask Deep red for 1 h at 4°C. Cells were then fixed in 4% paraformaldehyde and stained with an Apollo 488 or 567 stain kit (C10371-3 and C10371-1, RIBOBIO) at room temperature for 30 min. Images were captured on a Zeiss LSM 800 confocal microscope and processed with ImageJ software for quantitative image analysis.

### Viral attachment assays

Cells were pre-treated with DMSO, JQ-1 (1 μM), OTX-015 (10 μM) and I-BET 151 (10 μM) at 37°C for 24 h. Then cells were incubated with viruses at the indicated MOI and combined with DMSO, JQ-1 (1 μM), OTX-015 (10 μM) and I-BET 151 (10 μM) for 1 h at 4°C, then washed three times with ice cold PBS. Viral attachment assays were performed by RT-qPCR, immunoblotting and fluorescence analysis.

### Histological analysis

Tissues dissected from mice were fixed in 4% paraformaldehyde (158127, Sigma) overnight, embedded in paraffin, sectioned, and stained with hematoxylin and eosin solution.

### Statistical analysis

All data were analyzed in Prism 7 software (GraphPad Software, Inc) with two-tailed Student’s *t*-test or one-way ANOVA. *P* < 0.05 was considered statistically significant. For mouse survival studies, Kaplan-Meier survival curves were generated and analyzed for statistical significance.

## Supporting information

S1 Fig Effects of BRD4 inhibition on cell-cycle arrest and apoptosis. (a) Apoptosis was assessed with flow cytometry in Scramble, shBRD4-1 and shBRD4-2 PK15 cells. (b) Apoptosis was assessed with flow cytometry in HeLa and HEK293 cells treated with DMSO, JQ-1 (1 μM), OTX-015 (10 μM), I-BET 151 (10 μM) and wortmannin (2.5 μM) for 24 h. (c) Phospho-P53, total P53, MDM2 and P21 were assessed with immunoblotting analysis in HEK293 cells treated with DMSO, JQ-1 (1 μM), OTX-015 (10 μM) and I-BET 151 (10 μM) for 24 h. Actin served as a loading control.
(TIF)

S2 Fig BRD4 inhibitors attenuate viral infection. (a) Viral titer was assessed with TCID_50_ assays in PK15 cells infected with PRV-QXX (MOI = 0.1) and treated with JQ-1 (0–1000 nM), OTX-015 (0–10 μM) and I-BET 151 (0–10 μM) for 24 h. (b) Viral titer was assessed with TCID_50_ assays in A549 cells infected with HSV1-F (MOI = 1) and treated as in (a). (c) Viral titer was assessed with TCID_50_ assays in Vero cells infected with ECTV (MOI = 10) and treated with JQ-1 (0–1000 nM) for 24 h. (d) Viral titer was assessed with TCID_50_ assays in PK15 cells infected with VSV-GFP (MOI = 0.001) and treated as in (c). (e) Viral titer was assessed with TCID_50_ assay in MARC-145 cells infected with PRRSV-BJ4 (MOI = 1) and treated as in (c). (f and g) Viral titer was assessed with HA assays in Vero cells infected with NDV-GFP (f, MOI = 10), in MDCK infected with H1N1-PR8 (g, MOI = 1) and treated as in (c). All data are shown as mean ± SD based on three independent experiments. * P < 0.05, ** P < 0.01, *** P < 0.001 determined by two-tailed Student’s *t*-test.
(TIF)

S3 Fig BRD4 inhibition influences viral attachment. (a) PRV-QXX were incubated with DMSO, JQ-1 (1 μM), OTX-015 (10 μM) and I-BET 151 (10 μM) for 2 h at 37°C, and then dialysis was performed to remove the compounds. Viral attachment was assessed with RT-qPCR analysis in PK15 cells incubated with PRV-QXX (MOI = 1). (b) Viral attachment was assessed with immunofluorescence against PRV gE in PK15 cells incubated with PRV-QXX (MOI = 1). Data are shown as mean ± SD based on three independent experiments. ** P < 0.01 determined by two-tailed Student’s *t*-test. Scale bar, 10 μm.
(TIF)

S4 Fig JQ-1 activates cGAS/STING/TBK1-dependent innate immune responses. (a) IFN-β mRNA was assessed with RT-qPCR analysis in wild type (WT), STING^-/-^ and TBK1^-/-^ PK15 cells treated with JQ-1 (1 μM) at 0, 2, 4, 6, 12, 24 and 36 hpt. (b) IL-1β mRNA was assessed with RT-qPCR analysis in wild type (WT), STING^-/-^ and TBK1^-/-^ PK15 cells treated as in a. All data are shown as mean ± SD based on three independent experiments. * P < 0.05, ** P < 0.01, *** P < 0.001 determined by two-tailed Student’s *t*-test.
(TIF)
