# Supplementary figures and images for: BRD4 inhibition exerts anti-viral activity through DNA damage-dependent innate immune responses
Source: PLoS Pathog. 2020 Mar 24;16(3):e1008429. doi: 10.1371/journal.ppat.1008429 (PMC7122826; doi:10.1371/journal.ppat.1008429)

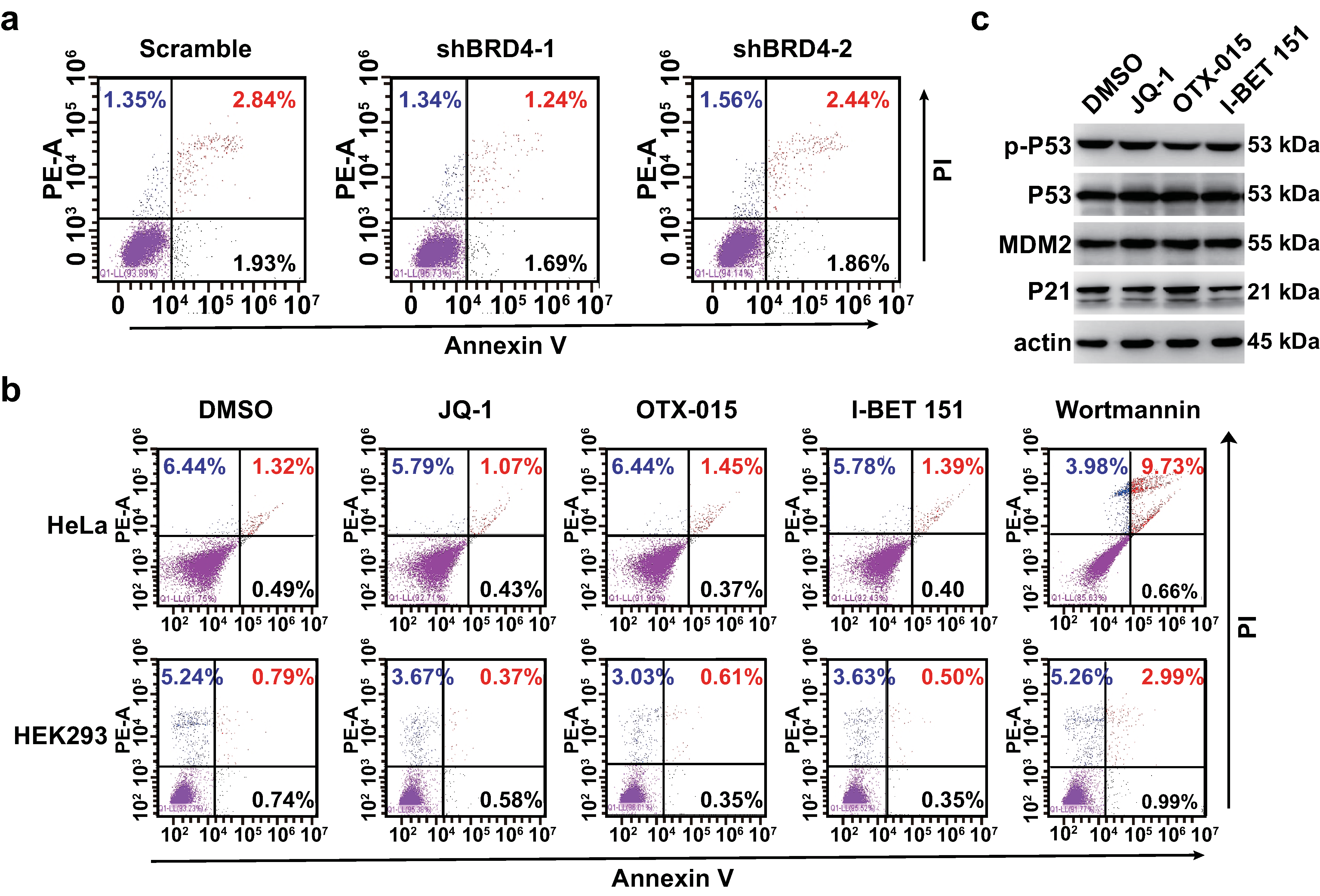

Supplement: S1 Fig — (a) Apoptosis was assessed with flow cytometry in Scramble, shBRD4-1 and shBRD4-2 PK15 cells. (b) Apoptosis was assessed with flow cytometry in HeLa and HEK293 cells treated with DMSO, JQ-1 (1 μM), OTX-015 (10 μM), I-BET 151 (10 μM) and wortmannin (2.5 μM) for 24 h. (c) Phospho-P53, total P53, MDM2 and P21 were assessed with immunoblotting analysis in HEK293 cells treated with DMSO, JQ-1 (1 μM), OTX-015 (10 μM) and I-BET 151 (10 μM) for 24 h. Actin served as a loading control. (TIF) [file ppat.1008429.s001.tif]

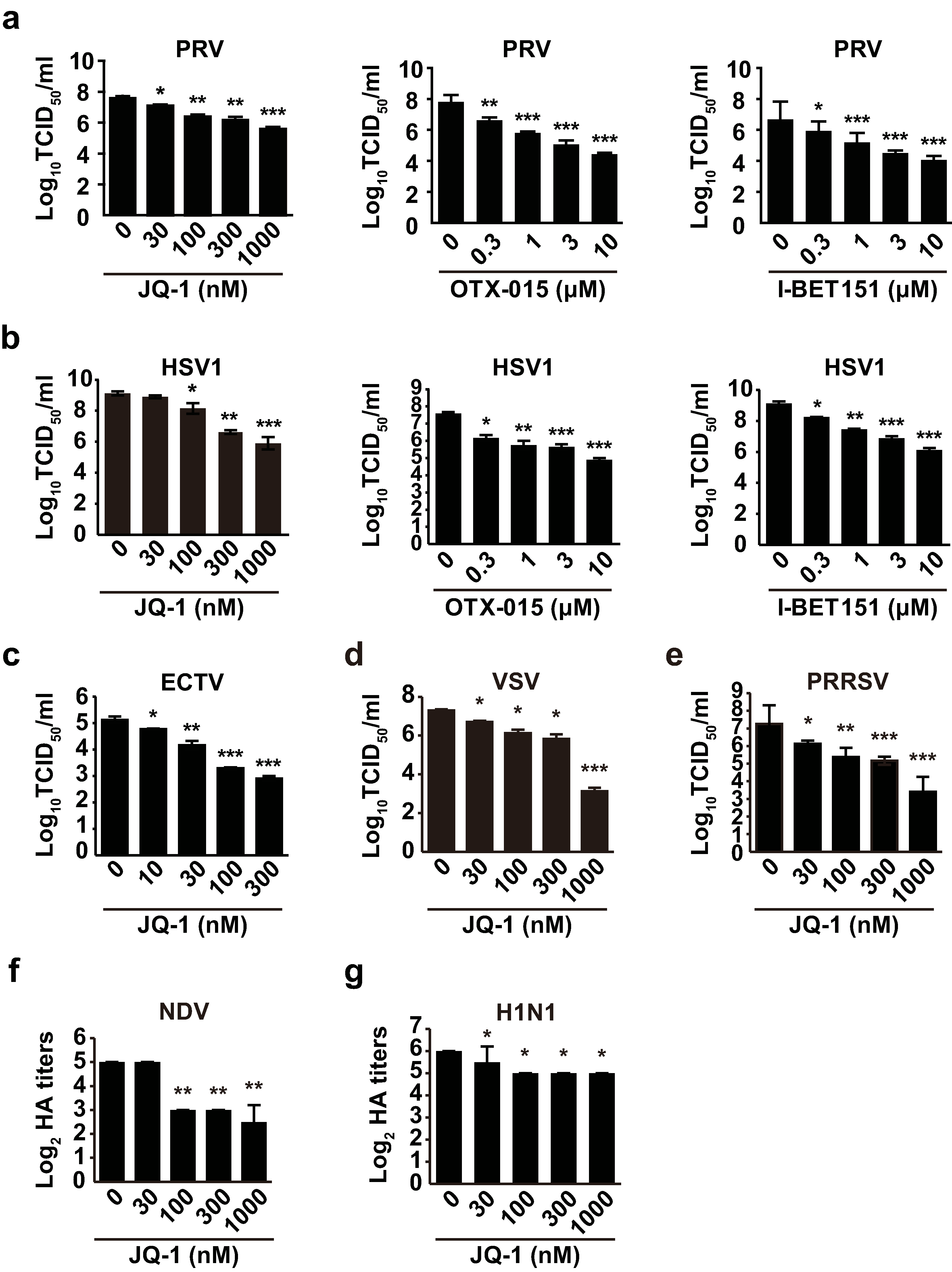

Supplement: S2 Fig — (a) Viral titer was assessed with TCID50 assays in PK15 cells infected with PRV-QXX (MOI = 0.1) and treated with JQ-1 (0–1000 nM), OTX-015 (0–10 μM) and I-BET 151 (0–10 μM) for 24 h. (b) Viral titer was assessed with TCID50 assays in A549 cells infected with HSV1-F (MOI = 1) and treated as in (a). (c) Viral titer was assessed with TCID50 assays in Vero cells infected with ECTV (MOI = 10) and treated with JQ-1 (0–1000 nM) for 24 h. (d) Viral titer was assessed with TCID50 assays in PK15 cells infected with VSV-GFP (MOI = 0.001) and treated as in (c). (e) Viral titer was assessed with TCID50 assay in MARC-145 cells infected with PRRSV-BJ4 (MOI = 1) and treated as in (c). (f and g) Viral titer was assessed with HA assays in Vero cells infected with NDV-GFP (f, MOI = 10), in MDCK infected with H1N1-PR8 (g, MOI = 1) and treated as in (c). All data are shown as mean ± SD based on three independent experiments. * P < 0.05, ** P < 0.01, *** P < 0.001 determined by two-tailed Student’s t-test. (TIF) [file ppat.1008429.s002.tif]

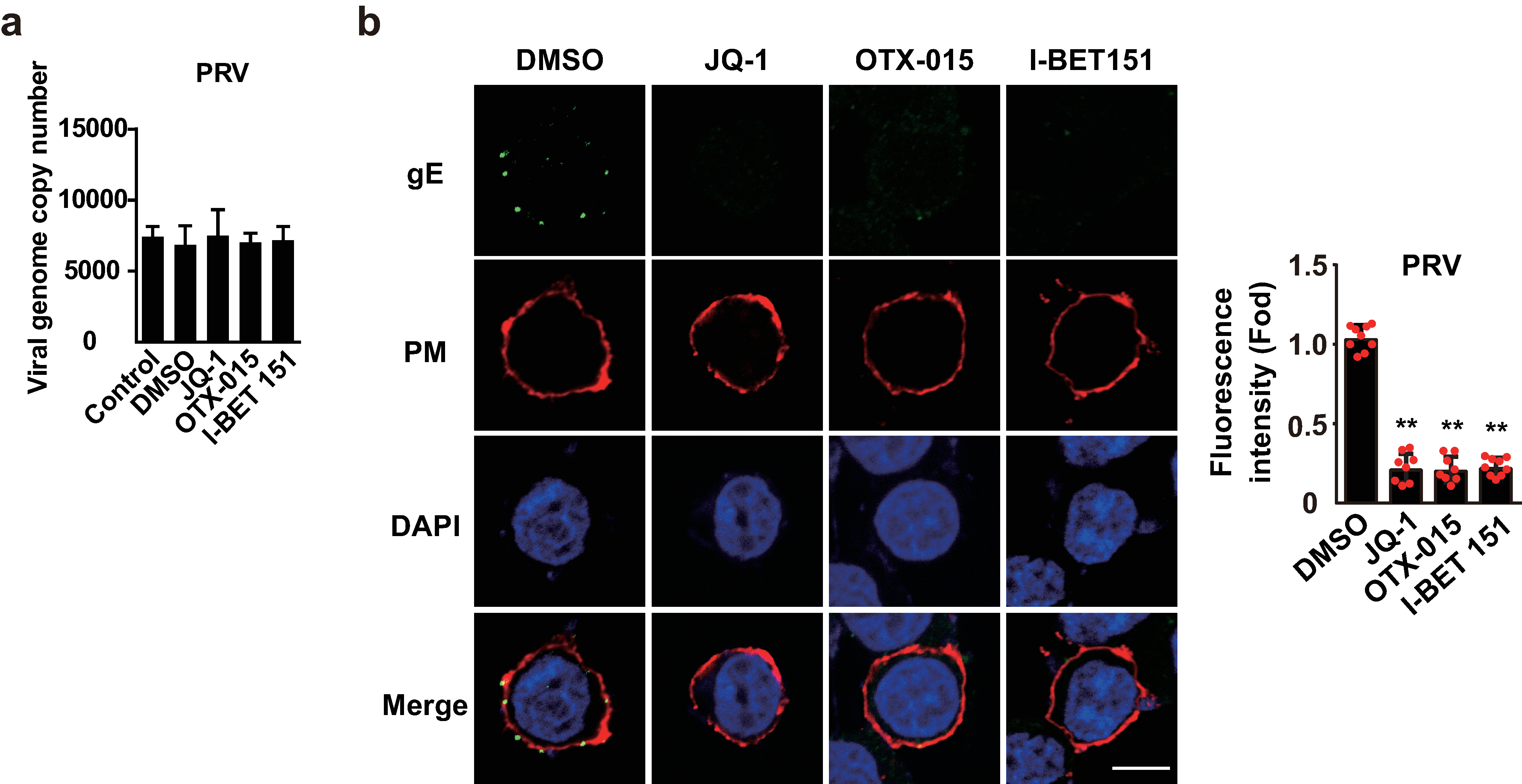

Supplement: S3 Fig — (a) PRV-QXX were incubated with DMSO, JQ-1 (1 μM), OTX-015 (10 μM) and I-BET 151 (10 μM) for 2 h at 37°C, and then dialysis was performed to remove the compounds. Viral attachment was assessed with RT-qPCR analysis in PK15 cells incubated with PRV-QXX (MOI = 1). (b) Viral attachment was assessed with immunofluorescence against PRV gE in PK15 cells incubated with PRV-QXX (MOI = 1). Data are shown as mean ± SD based on three independent experiments. ** P < 0.01 determined by two-tailed Student’s t-test. Scale bar, 10 μm. (TIF) [file ppat.1008429.s003.tif]

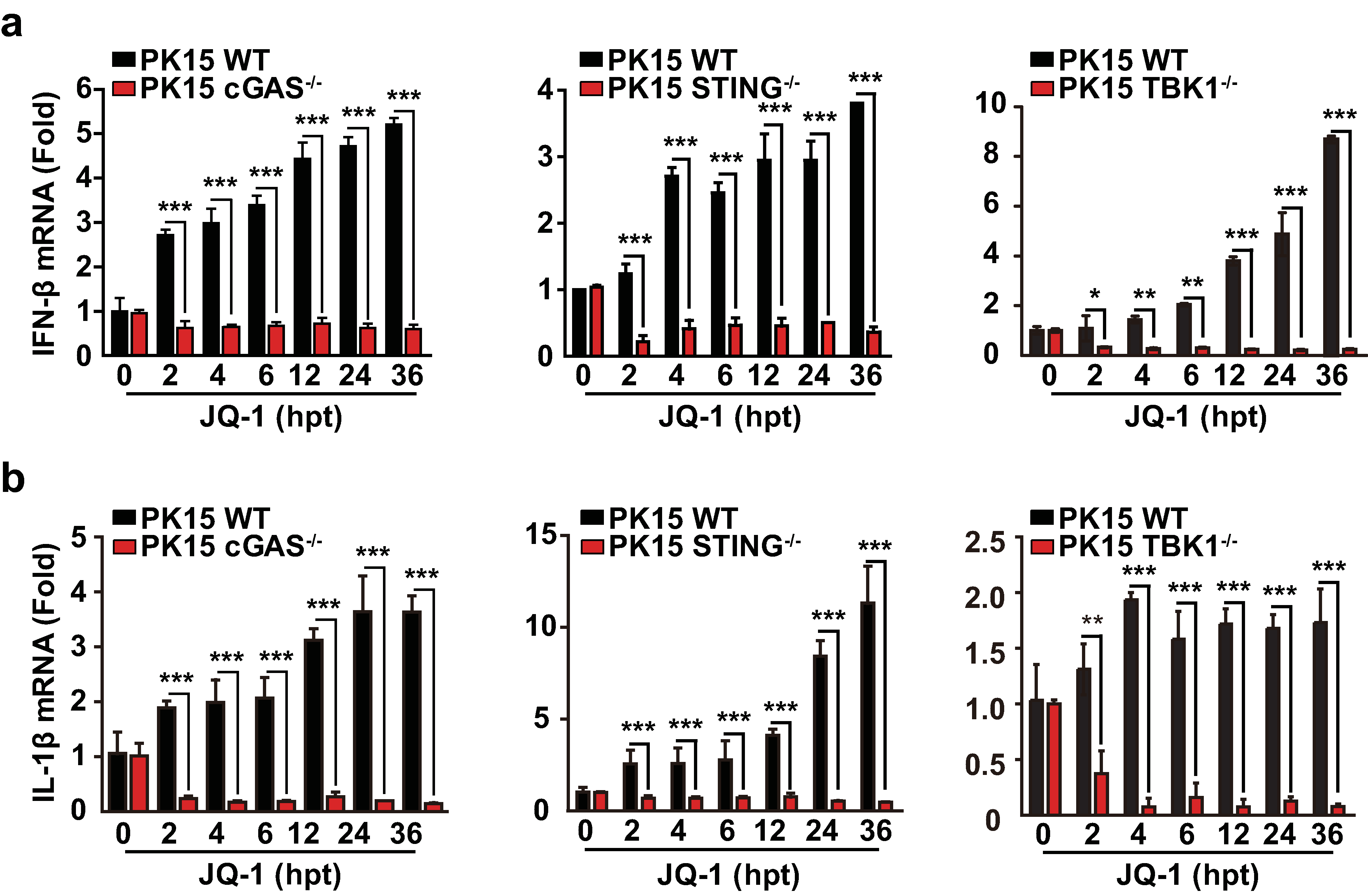

Supplement: S4 Fig — (a) IFN-β mRNA was assessed with RT-qPCR analysis in wild type (WT), STING-/- and TBK1-/- PK15 cells treated with JQ-1 (1 μM) at 0, 2, 4, 6, 12, 24 and 36 hpt. (b) IL-1β mRNA was assessed with RT-qPCR analysis in wild type (WT), STING-/- and TBK1-/- PK15 cells treated as in a. All data are shown as mean ± SD based on three independent experiments. * P < 0.05, ** P < 0.01, *** P < 0.001 determined by two-tailed Student’s t-test. (TIF) [file ppat.1008429.s004.tif]
